# Modeling Study of the Creep Behavior of Carbon-Fiber-Reinforced Composites: A Review

**DOI:** 10.3390/polym15010194

**Published:** 2022-12-30

**Authors:** Mostafa Katouzian, Sorin Vlase, Marin Marin, Maria Luminita Scutaru

**Affiliations:** 1 Department of Mechanical Engineering, Transilvania University of Brasov, 500036 Brasov, Romania; 2 Romanian Academy of Technical Sciences, 700506 Bucharest, Romania; 3 Department of Mathematics and Computer Science, Transilvania University of Brasov, 500036 Brasov, Romania

**Keywords:** FRP, composite, viscoelasticity, generalized Hooke law, creep response, constitutive low, carbon

## Abstract

The aim of this paper is to present some important practical cases in the analysis of the creep response of unidirectional fiber-reinforced composites. Some of the currently used models are described: the micromechanical model, homogenization technics, the Mori–Tanaka method, and the finite element method (FEM). Each method was analyzed to determine its advantages and disadvantages. Regarding the accuracy of the obtained results, comparisons are made with experimental tests. The methods presented here are applied to carbon-fiber-reinforced composites, but these considerations can also be applied to other types of composite materials.

## 1. Introduction

**Figure 1:**
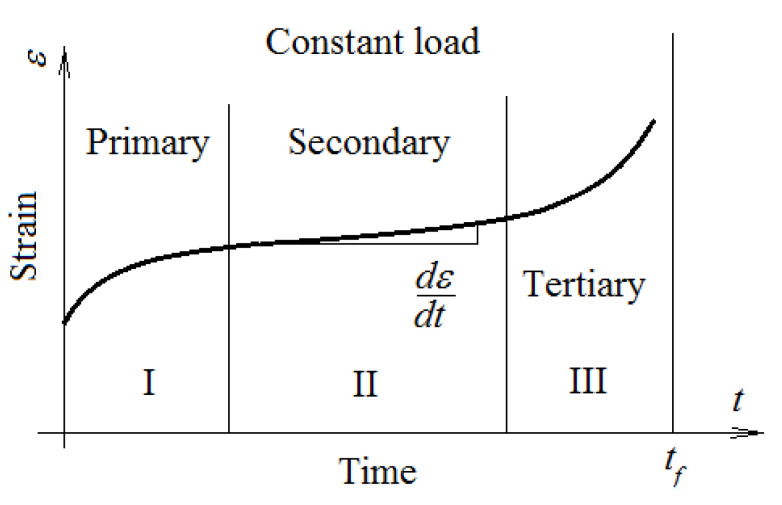
Usual creep behavior of a material (adapted from [2]).

The creep phenomenon that can occur in viscoelastic materials is defined as manifesting in three hypostases: primary, secondary, and tertiary. The creep phenomenon is defined as a deformation in time of the studied material, if it is loaded with a known force [1] (Figure 1). Creep phenomena usually manifest at high temperatures. However, there are situations in which the creep can appear at lower temperatures, for example, at room temperature, for some types of materials.

Of course, this phenomenon, which manifests in the elongation of the material over time, can become dangerous in the operation of a machine. Figure 1 shows the three intervals of creep behavior. Current applications refer mostly to the first two stages of creep, when the deformation rate is relatively high. In the primary creep stage, a high rate is observed at the beginning, which slows down over time. The aspect of the creep curve depends on the material, load, and time. In the secondary creep stage, there is a relatively constant rate. A high rate of deformation characterizes the third creep stage. The time interval in which this high increase is observed is short and is associated with the destruction of the material. In engineering practice, it is not necessary to reach this stage; as a result, the study of behavior in this area has not attracted much attention. Designers must know the rate of deformation. This can be determined using measurements or by using a verified calculus model. The paper presents such models, which are useful for design activities [2,3]. Creep behavior is interesting for engineers and studies on this phenomenon are numerous [4,5,6].

The technology of advanced composites has developed to the point where these materials are being increasingly utilized in the commercial, military, and aerospace industries, among others. Composite materials are ideal for structural applications where high strength-to-weight and stiffness-to-weight ratios, improved fatigue resistance, and improved dimensional stability are required. Reinforced fiber polymers date back to the early years of the last century. There are two major steps in the manufacturing of polymer-based laminated composites, namely layup and curing. In the layup stage, continuous filaments are arranged in unidirectional laminae or are interwoven. The fibers are often impregnated with resinous material, such as polyester resin, which later serves as the matrix material. The next step, thermal curing, involves the drying or polymerization of the resinous matrix material and is accomplished in suitable autoclaves. The aim is to form a permanent bond between the fibers and the matrix, as well as between the laminae, in order to obtain lightweight, stiff panels [2].

The materials used in engineering have different purposes and are manufactured according to different technologies; as a result, they have a variety of properties. The creep diagrams of these materials can be very different, even under the same loading and temperature conditions. The simplest way to construct a creep diagram is to perform experimental measurements. However, such an approach is expensive and time consuming. Loads with different constant loads must be considered, and tests must be performed at different temperatures.

In [7], a scheme for accelerated characterization is proposed to analyze the viscoelastic response of general laminated composites. The use of this scheme allows a small number of experimental measurements to be performed. The measurements allow for short-term tests at high temperature, to predict the long-term response [8,9,10].

It would be much more advantageous for designers to have useful creep models which could be used to obtain creep diagrams by calculation.

To study the nonlinear viscoelastic behavior of a unidirectional composite, the well-known FEM method is applied. The symmetry properties of the composite allow for the simplification of such an analysis. A good correlation with the FEM micromechanics models developed in [11] is obtained. The method can also be used to study a composite with a complex topology [12,13,14]. Such a description also offers the possibility of studying the material in a wide range of boundary conditions. Thus, the thermal effects and the expansion due to humidity were included through the initial conditions. In [15], the above equations were used for unidirectional composites reinforced with graphite and glass. 

The works [16,17,18] improve the classic models used in the case of nonlinear behavior. An empirical model was developed to achieve this. A method that can be easily implemented using a numerical procedure was thus obtained.

Based on the previously presented studies [16,17,18], a nonlinear viscoelastic model was developed in [19,20]. The developed model and the experimental measurements taken for test specimens allowed for an orthotropic material. The presented procedure can also be applied to study the long-term nonlinear viscoelastic response of laminates.

Other research [21] has shown that a law moisture concentration (at about 1%) can be a critical limit for carbon epoxy laminates. When this limit is exceeded, the viscoelastic rate of deformation occurs faster.

The study of a material made of an epoxy resin reinforced with unidirectional aramid fibers by tests and measurements at high temperatures is presented in [22]. An appropriate mathematical model for this study proved to be the “power law” which can describe behavior in both the linear and nonlinear domains, so that it can model viscoelastic behavior. To study the behavior in the nonlinear field, some nonlinear viscoelastic coefficients are introduced (these coefficients depend both on the stresses to which the materials are subjected and on the temperature). This method of analysis was proven to concur with the nonlinear model presented in [12].

In [23,24,25,26], a variational principle is used in which the time variable also appears, using a relatively simple mathematical description. In [27], the heat-induced stress field in the components of a polymer composite at low temperatures is studied (one application is considered for spacecraft). The geometry of the composite microstructure proves to be important in terms of the field of stresses and the deformation of this type of material under the conditions described above.

In [28], all the engineering constants that define one orthotropic and one transverse isotropic composite are determined. For a transverse isotropic material, the results [29,30,31,32] provide us with the upper and lower limits of engineering constants. In [33], the Mori–Tanaka method presented in [34] is extended.

Paper [35] shows the non-linear viscoelastic/viscoplastic behaviors of graphite/bismaleimide. Paper [12] presents a nonlinear formulation used to study materials at temperatures above 93 °C. A micromechanical analysis for the study of the behavior of a fiber-reinforced composite is described in [36,37]. These studies show good concordance with the findings presented in [11]. Biphasic materials and their mechanical properties have been extensively studied in numerous papers published in recent years [38,39,40,41,42,43,44,45,46,47,48,49]. New results are presented in [50,51,52,53,54].

In this review, the authors present more factors related to the analysis of the creep behavior of a composite material reinforced with fibers. The model’s proposed offer results and a creep curve in the case of different loads. The results presented in this review are mainly based on the results obtained in [55,56,57,58,59].

The creep calculation of composite materials represents an important step in the process of designing a new material. A series of methods are therefore developed to achieve this objective. The problem remains an important one in the context of unprecedented advances in the development of new materials, with increasing numbers of properties that are useful in various applications. To the knowledge of the authors, the systematization and unitary presentation of these methods has not yet been achieved. This study thus makes a significant contribution to the field. The methods based on the homogenization theory are presented in Section 2, Section 3 and Section 4, and those based on the FEM theory are presented in Section 5.

## 2. The Micromechanical Model in Homogenization Theory

### 2.1. Model and Constitutive Law

**Figure 2:**
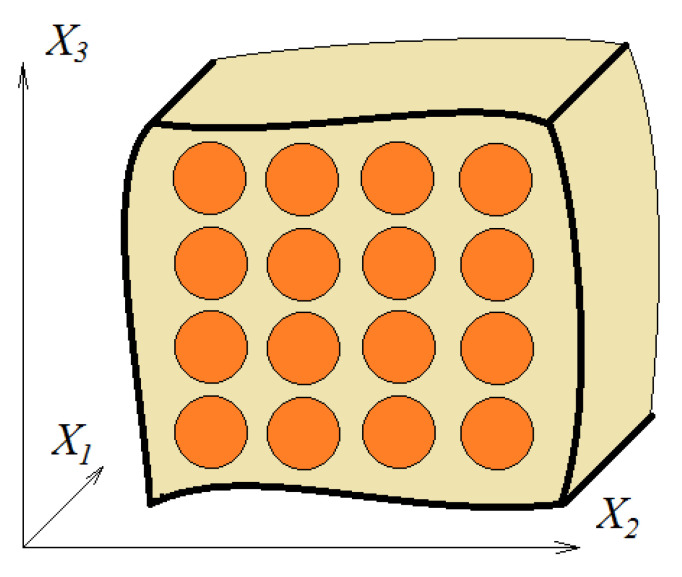
A micromechanical model of a one-dimensional fiber material.

The method of the micromechanical model aims to obtain the overall mechanical parameters of a composite based on models that use the parameters of the individual constituents of the composite and the interaction that exists between them. Consider a unidirectional composite with randomly distributed fibers in the matrix material. In the models used, it is normal to take into account a periodicity in the distribution of fibers. In this way, the existing periodicities allow us to simplify the analysis. Figure 2 presents such a model. The following assumptions can be formulated:

- The fibers are continuous and circular, and oriented in the X_1_ direction. They are positioned regularly in a rectangular array in the transversal X_2_–X_3_ plane;
- The fibers are linearly elastic and anisotropic. The matrix is isotropic and nonlinearly viscoelastic;
- No cracks or holes appear or develop, and the contact fiber matrix is mechanical.

**Figure 3:**
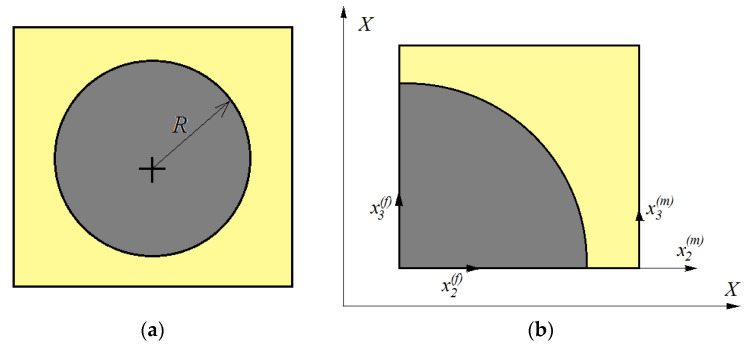
(**a**) A cell of the microstructure. (**b**) The representative unit cell (RUC).

Using the proposed model, it is possible to determine the response of the material if only a single repeating unit cell (RUC) is studied—see Figure 3. As such, the complexity of the problem can be significantly reduced. For this analysis, it is sufficient to study only a quarter of a fiber, as in Figure 3b. The main hypothesis of the theory is that the RUCs are very small, reported to be equal to the dimensions of the studied material.

**Figure 4:**
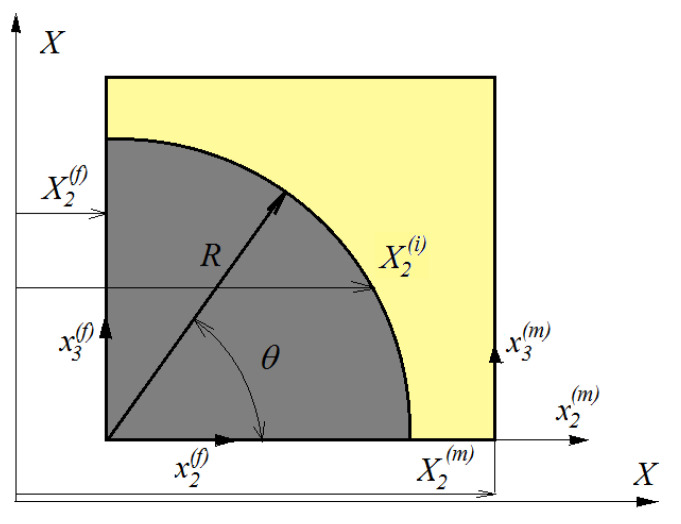
Continuity conditions in the RUC.

The RUC refers to a local coordinate system (*X*_1_, $x_{2}^{\left(\lambda \right)}$, $x_{3}^{\left(\lambda \right)}$) (Figure 4). The displacement in each subcell is defined through the following formulas [27,28]:

(1) $$u_{i}^{\left(\lambda \right)}=u_{i}^{o(\lambda )}+x_{2}^{\left(\lambda \right)}\xi_{i}^{\left(\lambda \right)}+x_{3}^{\left(\lambda \right)}\zeta_{i}^{\left(\lambda \right)}\;;\;i=1,2,3\;.$$

Here, $u_{i}^{o}$ is the displacement component of the origin and “λ” represents both the fiber (when λ=f) and the matrix (when λ=m).

Considering the material to be linear, the strain–displacement relations are as follows:

(2) $$\varepsilon_{ij}=\frac{1}{2}\left(\frac{\partial u_{i}}{\partial x_{j}}+\frac{\partial u_{j}}{\partial x_{i}}\right)\;;\;i,j=1,2,3\;,$$

Equation (2) can be written for fiber and matrix in the unified form:

(3) $$\varepsilon_{ij}^{\left(\lambda \right)}=\frac{1}{2}\left(\frac{\partial u_{i}^{\left(\lambda \right)}}{\partial x_{j}}+\frac{\partial u_{j}^{\left(\lambda \right)}}{\partial x_{i}}\right)\;;\;i,j=1,2,3$$

If i≠j , it can be written as

(4) $$\varepsilon_{ij}^{\left(\lambda \right)}=\frac{1}{2}\left(\frac{\partial u_{i}^{\left(\lambda \right)}}{\partial x_{j}}+\frac{\partial u_{j}^{\left(\lambda \right)}}{\partial x_{i}}\right)=\frac{\gamma_{ij}^{\left(\lambda \right)}}{2}\;;\;i,j=1,2,3\;;\;i\neq j$$

The engineering shear strain is denoted as $\gamma_{ij}^{\left(\lambda \right)}=2\varepsilon_{ij}^{\left(\lambda \right)}\;;\;i,j=1,2,3$ ; i≠j.

Using Equation (1) into Equation (2) and considering Equations (3) and (4), the following equations can be obtained:

(5) $$\varepsilon_{11}^{\left(\lambda \right)}=\frac{\partial u_{i}^{o(\lambda )}}{\partial X_{1}}\;$$

(6) $$\varepsilon_{11}^{\left(\lambda \right)}=\frac{\partial u_{i}^{o(\lambda )}}{\partial X_{1}}\;$$

(7) $$\varepsilon_{33}^{\left(\lambda \right)}=\zeta_{3}^{\left(\lambda \right)}$$

(8) $$\gamma_{23}^{\left(\lambda \right)}=\left[\xi_{3}^{\left(\lambda \right)}+\zeta_{2}^{\left(\lambda \right)}\right]$$

(9) $$\gamma_{31}^{\left(\lambda \right)}=\left[\frac{\partial u_{3}^{o(\lambda )}}{\partial X_{1}}+\zeta_{1}^{\left(\lambda \right)}\right]$$

(10) $$\gamma_{12}^{\left(\lambda \right)}=\left[\frac{\partial u_{2}^{o(\lambda )}}{\partial X_{1}}+\xi_{1}^{\left(\lambda \right)}\right]$$

Considering a linear and transversely isotropic composite, the constitutive equation can be written as

(11) $${\left\{\begin{array}{c} \varepsilon_{11}\\ \varepsilon_{22}\\ \varepsilon_{33}\\ \gamma_{23}\\ \gamma_{31}\\ \gamma_{12} \end{array}\right\}}^{\left(\lambda \right)}={\left[\begin{array}{cccccc} S_{11} & S_{12} & S_{12} & 0 & 0 & 0\\ S_{12} & S_{22} & S_{23} & 0 & 0 & 0\\ S_{12} & S_{23} & S_{22} & 0 & 0 & 0\\ 0 & 0 & 0 & S_{44} & 0 & 0\\ 0 & 0 & 0 & 0 & S_{66} & 0\\ 0 & 0 & 0 & 0 & 0 & S_{66} \end{array}\right]}^{\left(\lambda \right)}{\left\{\begin{array}{c} \sigma_{11}\\ \sigma_{11}\\ \sigma_{11}\\ \tau_{23}\\ \tau_{31}\\ \tau_{12} \end{array}\right\}}^{\left(\lambda \right)}$$

or, considering the expression of the engineering constant for this type of material:

(12) $${\left\{\begin{array}{c} \varepsilon_{11}\\ \varepsilon_{22}\\ \varepsilon_{33}\\ \gamma_{23}\\ \gamma_{31}\\ \gamma_{12} \end{array}\right\}}^{\left(\lambda \right)}={\left[\begin{array}{cccccc} \frac{1}{E_{11}} & -\frac{\nu_{12}}{E_{22}} & -\frac{\nu_{12}}{E_{22}} & 0 & 0 & 0\\ -\frac{\nu_{12}}{E_{22}} & \frac{1}{E_{22}} & -\frac{\nu_{23}}{E_{22}} & 0 & 0 & 0\\ -\frac{\nu_{12}}{E_{22}} & -\frac{\nu_{23}}{E_{22}} & \frac{1}{E_{22}} & 0 & 0 & 0\\ 0 & 0 & 0 & \frac{1}{G_{23}} & 0 & 0\\ 0 & 0 & 0 & 0 & \frac{1}{G_{12}} & 0\\ 0 & 0 & 0 & 0 & 0 & \frac{1}{G_{12}} \end{array}\right]}^{\left(\lambda \right)}{\left\{\begin{array}{c} \sigma_{11}\\ \sigma_{22}\\ \sigma_{33}\\ \tau_{23}\\ \tau_{31}\\ \tau_{12} \end{array}\right\}}^{\left(\lambda \right)}$$

Here, $E_{11}$ and $E_{22}=E_{33}$ are Young’s moduli, $G_{23}$ and $G_{12}=G_{13}$ are the shear moduli, and $\nu_{23}$ and $\nu_{12}=\nu_{13}$ are the Poisson ratios. The direction of anisotropy is, in our model, *X*_1_, and the plane of isotropy is *X*_2_*–X*_3_. From Equation (12), the following formula is obtained:

(13) $${\left\{\begin{array}{c} \sigma_{11}\\ \sigma_{22}\\ \sigma_{33}\\ \tau_{23}\\ \tau_{31}\\ \tau_{12} \end{array}\right\}}^{\left(f\right)}={\left[\begin{array}{cccccc} C_{11} & C_{12} & C_{12} & 0 & 0 & 0\\ C_{12} & C_{22} & C_{23} & 0 & 0 & 0\\ C_{12} & C_{23} & C_{22} & 0 & 0 & 0\\ 0 & 0 & 0 & C_{66} & 0 & 0\\ 0 & 0 & 0 & 0 & C_{44} & 0\\ 0 & 0 & 0 & 0 & 0 & C_{44} \end{array}\right]}^{\left(f\right)}{\left\{\begin{array}{c} \varepsilon_{11}\\ \varepsilon_{22}\\ \varepsilon_{33}\\ \gamma_{23}\\ \gamma_{31}\\ \gamma_{12} \end{array}\right\}}^{\left(f\right)}$$

with

(14) $$C_{66}=\frac{C_{22}-C_{23}}{2}$$

Equation (13) can be written as

(15) $$\sigma^{\left(f\right)}=C^{\left(f\right)}\varepsilon^{\left(f\right)}$$

The behavior of a viscoelastic material can be described using Boltzmann’s superposition principle and the results presented in [12]:

(16) $$\varepsilon (t)=D_{n}\;\sigma_{n}$$

with

(17) $$D_{n}=g_{o}D_{o}+g\sum_{j=1}^{m}D_{j}\left(1-e^{-t/r_{j}}\right)$$

The paper [12] presents us with the possibility of writing the constitutive equations as

(18) $$\varepsilon_{ij}^{\left(m\right)}=D_{n}\left[1+\nu (t)\right]\sigma_{ij}^{\left(m\right)}-D_{n}\;\nu (t)\;\sigma_{kk}^{\left(m\right)}\delta_{ij}$$

In Equation (18), $D_{n}$ is obtained using Equation (17), ν(t) is the Poisson ratio (in our study, this is considered to be independent of time), and $\delta_{ij}$ is Kronecker’s delta.

The first step must be to determine the average stresses, and then the average strains. Thus, the general behavior of the material is obtained based on the average stresses and average strains in a RUC.

### 2.2. Average Stress

In the proposed model, the RUC is considered to be a rectangular parallelepiped with parallel edges. The reference frame axes are (X_1_, X_2_, X_3_) of the volume V. This will be determined as the average stress ${\bar{\sigma }}_{ij}$ in V. This can be obtained via the following relation:

(19) $${\bar{\sigma }}_{ij}=\frac{1}{V}\int_{V}S_{ij}dV$$

Considering one-quarter of a cell, this relation can be written as

(20) $${\bar{\sigma }}_{ij}=\frac{1}{A}\left({\bar{S}}_{ij}^{\left(f\right)}A_{f}+{\bar{S}}_{ij}^{\left(m\right)}A_{m}\right)$$

where ${\bar{S}}_{ij}^{\left(\lambda \right)}$ are the average stresses. Now, consider a unit depth of the RUC, i.e., *V* = *A* × 1. Using the notation presented in Figure 3b, the following is obtained:

(21) $$A={\left(R+h/2\right)}^{2};\;A_{f}^{}=\pi R^{2}/4;\;A_{m}={\left(R+h/2\right)}^{2}-\pi R^{2}/4$$

(22) $${\bar{\sigma }}_{ij}=\frac{1}{{\left(R+\frac{h}{2}\right)}^{2}}\left\{\frac{\pi R^{2}}{4}{\bar{S}}_{ij}^{\left(f\right)}+\left[{\left(R+\frac{h}{2}\right)}^{2}-\frac{\pi R^{2}}{4}\right]{\bar{S}}_{ij}^{\left(m\right)}\right\}$$

The partial average stress ${\bar{S}}_{ij}^{\left(\lambda \right)}$ is obtained with

(23) $${\bar{S}}_{ij}^{\left(\lambda \right)}=\frac{1}{A_{\nu }}\int_{A}\sigma_{ij}^{\left(\lambda \right)}dA=\frac{1}{A_{\nu }}\iint \sigma_{ij}^{\left(\lambda \right)}dx_{2}dx_{3}$$

Using polar coordinates, the Jacobian can be obtained:

(24) $$J=\frac{\partial (x_{2},x_{3})}{\partial (r,\theta )}=\left|\begin{array}{cc} \cos\theta & \sin\theta \\ -r\sin\theta & r\cos\theta \end{array}\right|=r$$

Moreover, Equation (23) for fiber (subcell “*f*”) becomes

(25) $$e{\bar{S}}_{ij}^{\left(f\right)}=\frac{4}{\pi R^{2}}\int_{0}^{\pi /2}\int_{0}^{R}\sigma_{ij}^{\left(f\right)}rdrd\theta$$

where $\sigma_{ij}^{\left(f\right)}$ is given by Equation (13). Introducing Equations (5)–(10) and (11) into Equation (25) leads to

(26) $${\bar{S}}_{ij}^{\left(f\right)}=\left\{\begin{array}{c} C_{11}\varepsilon_{11}^{\left(f\right)}+C_{12}\left(\varepsilon_{22}^{\left(f\right)}+\varepsilon_{33}^{\left(f\right)}\right)\\ C_{12}\varepsilon_{11}^{\left(f\right)}+C_{22}\varepsilon_{22}^{\left(f\right)}+C_{23}\varepsilon_{33}^{\left(f\right)}\\ C_{12}\varepsilon_{11}^{\left(f\right)}+C_{23}\varepsilon_{22}^{\left(f\right)}+C_{22}\varepsilon_{33}^{\left(f\right)}\\ C_{66}\gamma_{23}^{\left(f\right)}\\ C_{44}\gamma_{31}^{\left(f\right)}\\ C_{44}\gamma_{12}^{\left(f\right)} \end{array}\right\}$$

or

(27) $${\bar{S}}_{ij}^{\left(f\right)}=\left\{\begin{array}{c} C_{11}\frac{\partial u_{1}^{o(f)}}{\partial X_{1}}+C_{12}\left(\xi_{2}^{\left(f\right)}+\zeta_{3}^{\left(f\right)}\right)\\ C_{12}\frac{\partial u_{1}^{o(f)}}{\partial X_{1}}+C_{22}\xi_{2}^{\left(f\right)}+C_{23}\zeta_{3}^{\left(f\right)}\\ C_{12}\frac{\partial u_{1}^{o(f)}}{\partial X_{1}}+C_{23}\xi_{2}^{\left(f\right)}+C_{22}\zeta_{3}^{\left(f\right)}\\ C_{66}\left[\xi_{3}^{\left(f\right)}+\zeta_{2}^{\left(f\right)}\right]\\ C_{44}\left[\frac{\partial u_{3}^{o(f)}}{\partial X_{1}}+\zeta_{1}^{\left(f\right)}\right]\\ C_{44}\left[\frac{\partial u_{2}^{o(f)}}{\partial X_{1}}+\xi_{1}^{\left(f\right)}\right] \end{array}\right\}$$

The average stresses in the matrix (subcell “*m*”) are determined using the following relations:

(28) $${\bar{S}}_{ij}^{\left(m\right)}=\frac{1}{{\left(R+\frac{h}{2}\right)}^{2}-\frac{\pi R^{2}}{4}}\left(\int_{0}^{R+h/2}\int_{0}^{R+h/2}\sigma_{ij}^{\left(m\right)}dx_{2}dx_{3}-\int_{0}^{\pi /2}\int_{0}^{R}\sigma_{ij}^{\left(m\right)}rdrd\theta \right)$$

Equation (15) together with Equations (5)–(10) yields

(29) $$\frac{\partial u_{1}^{o}}{\partial X_{1}}=D_{n}\left[1+\nu (t)\right]{\bar{S}}_{11}^{\left(m\right)}-D_{n}\left[\nu (t)\right]{\bar{S}}_{kk}^{\left(m\right)}$$

(30) $$\xi_{2}^{\left(m\right)}=D_{n}\left[1+\nu (t)\right]{\bar{S}}_{22}^{\left(m\right)}-D_{n}\left[\nu (t)\right]{\bar{S}}_{kk}^{\left(m\right)}$$

(31) $$\zeta_{3}^{\left(m\right)}=D_{n}\left[1+\nu (t)\right]{\bar{S}}_{33}^{\left(m\right)}-D_{n}\left[\nu (t)\right]{\bar{S}}_{kk}^{\left(m\right)}$$

(32) $$\xi_{3}^{\left(m\right)}+\zeta_{2}^{\left(m\right)}=2D_{n}\left[1+\nu (t)\right]{\bar{S}}_{23}^{\left(m\right)}$$

(33) $$\frac{\partial u_{3}^{o(m)}}{\partial X_{1}}+\zeta_{1}^{\left(m\right)}=2D_{n}\left[1+\nu (t)\right]{\bar{S}}_{31}^{\left(m\right)}$$

(34) $$\frac{\partial u_{2}^{o(m)}}{\partial X_{1}}+\xi_{1}^{\left(m\right)}=2D_{n}\left[1+\nu (t)\right]{\bar{S}}_{12}^{\left(m\right)}$$

### 2.3. Continuity Conditions

In a RUC, the conditions of continuity of the movements at the interface between sub-cells must be ensured. These conditions must be assured in both the *X*_2_ and *X*_3_ directions. From Figure 4, the following relations hold:

(35) $$x_{2}^{\left(f\right)}=X_{2}^{i}\mp X_{2}^{\left(f\right)}=\mp R\cos\theta$$

(36) $$x_{2}^{\left(m\right)}=X_{2}^{i}\mp X_{2}^{\left(m\right)}=\pm \left(\frac{h}{2}+R-R\cos\theta \right)$$

where there are θ located points on the interface.

Introducing Equations (35) and (36) into Equation (1) for the cases when λ=f and λ=m results in

(37) $$u_{i}^{\left(f\right)}=u_{i}^{o(f)}\mp R\cos\theta \xi_{i}^{\left(f\right)}+x_{3}^{\left(f\right)}\zeta_{i}^{\left(f\right)}$$

(38) $$u_{i}^{\left(m\right)}=u_{i}^{o(m)}\pm \left(\frac{h}{2}+R-R\cos\theta \right)\xi_{i}^{\left(m\right)}+x_{3}^{\left(m\right)}\zeta_{i}^{\left(m\right)}$$

where

(39) $$u_{i}^{o(f)}=u_{i}^{o(f)}(X_{2}^{\left(f\right)})$$

(40) $$u_{i}^{o(m)}=u_{i}^{o(m)}(X_{2}^{\left(m\right)})$$

The continuity of the displacements at the interface is considered in the average sense. This is expressed by the following relations:

$$\int_{-\pi /2}^{\pi /2}\left[u_{i}^{o(f)}\mp R\cos\theta \xi_{i}^{\left(f\right)}+x_{3}^{\left(f\right)}\zeta_{i}^{\left(f\right)}\right]R\cos\theta d\theta =$$

(41) $$\int_{-\pi /2}^{\pi /2}\left[u_{i}^{o(m)}\pm \left(\frac{h}{2}+R-R\cos\theta \right)\xi_{i}^{\left(m\right)}+x_{3}^{\left(m\right)}\zeta_{i}^{\left(m\right)}\right]R\cos\theta d\theta$$

Equation (41) produces

(42) $$u_{i}^{o(f)}\left(X_{2}^{\left(f\right)}\right)\pm \frac{\pi R}{2}\xi_{i}^{\left(f\right)}=u_{i}^{o(m)}\left(X_{2}^{\left(m\right)}\right)\mp \left(h+2R-\frac{\pi R}{2}\right)\xi_{i}^{\left(m\right)}$$

The addition of the two Equations (42) offers us

(43) $$u_{i}^{o(f)}=u_{i}^{o(m)}=u_{i}^{o}$$

which represent the continuity conditions for displacement.

### 2.4. Average Strain

Considering the composite specimen presented earlier, and the continuity conditions, the average of the strains over volume is

(44) $${\bar{\varepsilon }}_{ij}=\frac{1}{V}\int_{V}\varepsilon_{ij}dV$$

For the representative cell studied, the following formula is obtained:

(45) $${\bar{\varepsilon }}_{ij}=\frac{1}{A}\sum_{\lambda =f,m}{\bar{\varepsilon }}_{ij}^{\left(\lambda \right)}A_{\lambda }$$

Here $A=A_{m}+A_{f}$ and ${\bar{\varepsilon }}_{ij}^{\left(\lambda \right)}$ are the strains obtained using Equations (5)–(10) (λ=f,m). Considering Equations (1) and (3) (considering i=j=1), we obtain

(46) $${\bar{\varepsilon }}_{ij}^{\left(\lambda \right)}=\frac{\partial u_{1}^{o}}{\partial X_{1}}$$

Equation (46) together with Equation (45) yields

(47) $${\bar{\varepsilon }}_{11}=\frac{1}{{\left(R+\frac{h}{2}\right)}^{2}}\left\{\frac{\pi R^{2}}{4}\frac{\partial u_{1}^{o}}{\partial X_{1}}+\left[{\left(R+\frac{h}{2}\right)}^{2}-\frac{\pi R^{2}}{4}\right]\frac{\partial u_{1}^{o}}{\partial X_{1}}\right\}$$

or

(48) $${\bar{\varepsilon }}_{11}=\frac{\partial u_{1}^{o}}{\partial X_{1}}$$

The octahedral shear stress in the matrix is

(49) $$S_{oct}^{\left(m\right)}={\left\{\frac{1}{2}\left[{\left({\bar{S}}_{11}^{\left(m\right)}-{\bar{S}}_{22}^{\left(m\right)}\right)}^{2}+{\left({\bar{S}}_{22}^{\left(m\right)}-{\bar{S}}_{33}^{\left(m\right)}\right)}^{2}+{\left({\bar{S}}_{33}^{\left(m\right)}-{\bar{S}}_{11}^{\left(m\right)}\right)}^{2}\right]+3\left[{\left({\bar{S}}_{12}^{\left(m\right)}\right)}^{2}+{\left({\bar{S}}_{23}^{\left(m\right)}\right)}^{2}+{\left({\bar{S}}_{31}^{\left(m\right)}\right)}^{2}\right]\right\}}^{\frac{1}{2}}$$

The unknowns are calculated using an incremental procedure. The unknowns are

- the six values of stresses in the two subcells: ${\bar{S}}_{11}^{\left(\lambda \right)}$, ${\bar{S}}_{22}^{\left(\lambda \right)}$, and ${\bar{S}}_{33}^{\left(\lambda \right)}$;
- the four micro-variables in the two subcells: $\xi_{2}^{\left(\lambda \right)}$ and $\zeta_{3}^{\left(\lambda \right)}$;
- the three strains in the composite: ${\bar{\varepsilon }}_{11}$, ${\bar{\varepsilon }}_{22}$, and ${\bar{\varepsilon }}_{33}$.

Now, the connection between the stress in the matrix and in the fiber of a RUC must be determined. Using the assumptions proposed in [36,37], the shear stress in the *X*_2_ direction is

(50) $${\bar{S}}_{22}^{\left(f\right)}=\alpha_{f}{\bar{\sigma }}_{22}$$

for the fiber and

(51) $${\bar{S}}_{22}^{\left(m\right)}=\alpha_{m}{\bar{\sigma }}_{22}$$

for the matrix from where it results:

(52) $${\bar{S}}_{22}^{\left(f\right)}=\frac{\alpha_{f}}{\alpha_{m}}{\bar{S}}_{22}^{\left(m\right)}$$

In the X_3_ direction, a similar equation is obtained:

(53) $${\bar{S}}_{33}^{\left(f\right)}=\frac{\beta_{f}}{\beta_{m}}{\bar{S}}_{33}^{\left(m\right)}$$

The concentration factors $\alpha_{\lambda }$ and $\beta_{\lambda }$ are weighting coefficients and should satisfy the following relations:

(54) $$\alpha_{f}v_{f}+\alpha_{m}v_{m}=1$$

and

(55) $$\beta_{f}v_{f}+\beta_{m}v_{m}=1$$

In a particular case, considering that the composite loaded is in only one of the directions X_2_ or X_3_, the relation (38) in direction X_3_ (unloaded) becomes

(56) $${\bar{S}}_{33}^{\left(f\right)}=-\frac{v_{m}}{v_{f}}{\bar{S}}_{33}^{\left(m\right)}$$

and

(57) $${\bar{S}}_{22}^{\left(f\right)}=-\frac{v_{m}}{v_{f}}{\bar{S}}_{22}^{\left(m\right)}$$

Consider now the case of a uniaxial load. Therefore, we obtain a linear system with 13 equations and 13 unknowns:

(58) $${\bar{S}}_{11}^{\left(f\right)}=C_{11}{\bar{\varepsilon }}_{11}+C_{12}\left(\xi_{2}^{\left(f\right)}+\zeta_{3}^{\left(f\right)}\right)$$

(59) $${\bar{S}}_{22}^{\left(f\right)}=C_{12}{\bar{\varepsilon }}_{11}+C_{22}\xi_{2}^{\left(f\right)}+C_{23}\zeta_{3}^{\left(f\right)}$$

(60) $${\bar{S}}_{33}^{\left(f\right)}=C_{12}{\bar{\varepsilon }}_{11}+C_{23}\xi_{2}^{\left(f\right)}+C_{22}\zeta_{3}^{\left(f\right)}$$

(61) $${\bar{\varepsilon }}_{11}=D_{n}\left[1+\nu (t)\right]{\bar{S}}_{11}^{\left(m\right)}-D_{n}\left[\nu (t)\right]{\bar{S}}_{kk}^{\left(m\right)}$$

(62) $$\xi_{2}^{\left(m\right)}=D_{n}\left[1+\nu (t)\right]{\bar{S}}_{22}^{\left(m\right)}-D_{n}\left[\nu (t)\right]{\bar{S}}_{kk}^{\left(m\right)}$$

(63) $$\zeta_{3}^{\left(m\right)}=D_{n}\left[1+\nu (t)\right]{\bar{S}}_{33}^{\left(m\right)}-D_{n}\left[\nu (t)\right]{\bar{S}}_{kk}^{\left(m\right)}$$

(64) $${\bar{\varepsilon }}_{22}=\frac{1}{A}\left[A_{f}\xi_{2}^{\left(f\right)}+A_{m}\xi_{2}^{\left(m\right)}\right]$$

(65) $${\bar{\varepsilon }}_{33}=\frac{1}{A}\left[A_{f}\zeta_{3}^{\left(f\right)}+A_{m}\zeta_{3}^{\left(m\right)}\right]$$

(66) $${\bar{S}}_{22}^{\left(f\right)}=\frac{\alpha_{f}}{\alpha_{m}}{\bar{S}}_{22}^{\left(m\right)}$$

(67) $${\bar{S}}_{33}^{\left(f\right)}=\frac{\beta_{f}}{\beta_{m}}{\bar{S}}_{33}^{\left(m\right)}$$

(68) $${\bar{\sigma }}_{11}=\frac{1}{A}\left[A_{f}{\bar{S}}_{11}^{\left(f\right)}+A_{m}{\bar{S}}_{11}^{\left(m\right)}\right]$$

(69) $${\bar{\sigma }}_{22}=\frac{1}{A}\left[A_{f}{\bar{S}}_{22}^{\left(f\right)}+A_{m}{\bar{S}}_{22}^{\left(m\right)}\right]$$

(70) $${\bar{\sigma }}_{33}=\frac{1}{A}\left[A_{f}{\bar{S}}_{33}^{\left(f\right)}+A_{m}{\bar{S}}_{33}^{\left(m\right)}\right]$$

The analysis presented in this section shows that a micromechanical model for the study of a unidirectional composite can provide good results. Thus, analytical relations are obtained, which then allow for the calculation of the mechanical constants of such a composite and for the study of its behavior in a range of applications. Schapery’s nonlinear constitutive equation for isothermal uniaxial loading conditions is used in the analysis, thus allowing us to consider the nonlinear viscoelastic response of the material. Papers that present many experimental results [55,56,57,58,59] demonstrate the potential of the method.

## 3. Description of Homogenization Theory

### 3.1. Overview

The theory of homogenization is a mathematical method used to average the physical properties of inhomogeneous materials. This method has been developed over the last eight decades and is used to analyze and solve differential equations with periodic coefficients. As essentially inhomogeneous materials that have a periodicity or certain symmetries in their structure, composite materials lend themselves very well to the application of these methods that determine the mechanical characteristics of a material. The experimental results validate the methods used by the theory of homogenization [55,56,57,58,59]. For this reason, the homogenization method has been used in numerous cases and engineering applications [33,34,36,37,39,40,41,59] to determine the mechanical properties of multiphase composites. In this method, a transition is made, through homogenization, from a periodic structure to a homogeneous and isotropic or transversely isotropic material throughout its structure [44].

In the research, several analytical and numerical methods have been proposed to solve the problems generated by the application of this method. Experimental results have always shown the predominant acceptance of such methods [45,60]. The interaction between the phases of the composite is modeled by unifying the homogenization problems for heterogeneous elasto-plastic and elasto-viscoplastic materials [61,62]. Other works address the improvement of the method, using the experience gained by different engineering applications [61,62,63,64,65,66,67,68,69]. Other related methods are considered in [70,71]. The object of this research is the development of reliable procedures that can be easily applied by designers. The following section presents the homogenization theory used to determine the mechanical quantities that characterize the viscoelastic material in question. An application is suggested for a composite reinforced with carbon fibers.

### 3.2. Homogenized Model

One of the advantages offered by the homogenization theory is the possibility of studying differential equations in which the coefficients have rapid variations or periodic variations. Engineering constants, which are useful in engineering practice, are obtained following averaging processes. Thus, a material with a periodic structure can be treated as a homogeneous material. A differential equation with periodic coefficients with large variations is thus replaced in the modeling with an equation with constant coefficients. This is how the continuum concept is extended to micro-structured materials (composite materials also belong to this class). The bases of this mathematical theory are presented in [72,73,74,75,76,77]. In this application, the calculation method is used to analyze the creep response of a unidirectional composite reinforced with carbon fibers.

The stress field $\sigma^{\delta }$ for repeating unit cells of size δ must obey the following equations:

(71) $$\frac{\partial \sigma_{11}^{\delta }}{\partial x_{1}}+\frac{\partial \tau_{12}^{\delta }}{\partial x_{2}}+\frac{\partial \tau_{13}^{\delta }}{\partial x_{3}}=f_{1}(x)\;\frac{\partial \tau_{21}^{\delta }}{\partial x_{1}}+\frac{\partial \sigma_{22}^{\delta }}{\partial x_{2}}+\frac{\partial \tau_{23}^{\delta }}{\partial x_{3}}=f_{2}(x)\;\frac{\partial \tau_{31}^{\delta }}{\partial x_{1}}+\frac{\partial \tau_{32}^{\delta }}{\partial x_{2}}+\frac{\partial \sigma_{33}^{\delta }}{\partial x_{3}}=f_{3}(x)$$

where $\sigma_{ij}^{\delta }=\sigma_{ji}^{\delta }$, for i,j=1,2,3.

The contour conditions that must be respected by the displacements are

(72) $${u^{\delta }|}_{\partial_{1}\Omega }=\tilde{u}$$

The boundary conditions are

(73) $$\sigma_{11}^{\delta }n_{1}+\tau_{12}^{\delta }n_{2}+\tau_{13}^{\delta }n_{3}=T_{1}(x)\;\tau_{21}^{\delta }n_{1}+\sigma_{22}^{\delta }n_{2}+\tau_{23}^{\delta }n_{3}=T_{2}(x)\;\tau_{31}^{\delta }n_{1}+\tau_{32}^{\delta }n_{2}+\sigma_{33}^{\delta }n_{3}=T_{3}(x)$$

on the contour $\partial_{2}\Omega$, ($\partial_{1}\Omega \cup \partial_{2}\Omega =\partial \Omega$). Hook’s Law is

(74) $${\left\{\begin{array}{c} \sigma_{11}\\ \sigma_{22}\\ \sigma_{33}\\ \tau_{23}\\ \tau_{31}\\ \tau_{12} \end{array}\right\}}^{\delta }=\left[\begin{array}{cccccc} C_{1111} & C_{1122} & C_{1133} & & & \\ C_{2211} & C_{2222} & C_{2233} & & 0 & \\ C_{3311} & C_{3322} & C_{3333} & & & \\ & & & C_{2323} & & \\ & 0 & & & C_{3131} & \\ & & & & & C_{1212} \end{array}\right]{\left\{\begin{array}{c} \varepsilon_{11}\\ \varepsilon_{22}\\ \varepsilon_{33}\\ \gamma_{23}\\ \gamma_{31}\\ \gamma_{13} \end{array}\right\}}^{\delta }$$

Or, using a compact notation,

(75) $$\sigma^{\delta }=C\varepsilon^{\delta }$$

The elasticity matrix *C* is semi-positive definite:

(76) $$C_{ijkh}x_{ij}x_{kh}\geq \alpha \;x_{ij}x_{kh}$$

for α>0 and $\forall x_{ij},x_{kh}\in R$, $C_{ijkh}(x)$ is a periodical function of *x* with the period equal with the dimension δ of the unit cell. Considering a new function y, y=x/δ:

(77) $$C_{ijkh}(x)=C_{ijkh}(y\delta )=C_{ijkh}(y)$$

and the stress is

(78) $$\sigma_{ij}^{\delta }=\sigma_{ij}^{o}(x,y)+\sigma_{ij}^{1}(x,y)\delta +.....$$

The dependence of stress on *y* is “quasi-periodical”. Introducing Equation (78) in Equation (71) produces

(79) $$\delta^{-1}\frac{\partial \sigma_{ij}^{o}}{\partial y_{j}}+\left(\frac{\partial \sigma_{ij}^{o}}{\partial x_{j}}+\frac{\partial \sigma_{ij}^{1}}{\partial y_{j}}\right)\;\delta^{o}+\left(\frac{\partial \sigma_{ij}^{1}}{\partial x_{j}}+\frac{\partial \sigma_{ij}^{2}}{\partial y_{j}}\right)\;\delta^{1}+.....=f_{i}(x)$$

The following relation is used:

(80) $$\frac{d}{dx}\left(f\right)=\frac{\partial f}{\partial x}dx+\frac{\partial f}{\partial x}dy$$

but y=x/δ, and, thus, dy=dx/δ; so,

(81) $$\frac{d}{dx}\left(f\right)=\frac{\partial f}{\partial x}\left(f\right)+\frac{1}{\delta }\frac{\partial }{\partial y}\left(f\right)$$

The coefficients of $\delta^{-1}$ in Equation (79) must be 0; therefore,

(82) $$\frac{\partial \sigma_{ij}^{o}}{\partial y_{j}}=0$$

Equation (82) is called the “local equation”. Identifying the terms of $\delta {\;}^{0}$ produces

(83) $$\frac{\partial \sigma_{ij}^{o}}{\partial x_{j}}+\frac{\partial \sigma_{ij}^{1}}{\partial y_{j}}=f_{i}(x)\;i=1,2,3$$

Applying the average operator to Equation (83) results in the following equation:

(84) $$\frac{\partial \langle \sigma_{ij}^{o}\rangle }{\partial x_{j}}+\langle \frac{\partial \sigma_{ij}^{1}}{\partial y_{j}}\rangle =f_{i}(x)\;i=1,2,3$$

but

(85) $$\langle \frac{\partial \sigma_{ij}^{1}}{\partial y_{j}}\rangle =\frac{1}{V}\int_{V}\partial \sigma_{ij}^{1}dV=\frac{1}{V}\int_{\partial V}\sigma_{ij}^{1}n_{j}dS=0$$

The stresses $\sigma_{ij}^{1}$ take equal values on the corresponding points of the boundary of the cell Γ (due to the property of periodicity), so

(86) $$\frac{\partial \langle \sigma_{ij}^{o}\rangle }{\partial x_{j}}=f_{i}(x)\;i=1,2,3$$

We state that

(87) $$\varepsilon_{ij,x}(w)=\frac{1}{2}\left(\frac{\partial w_{i}}{\partial x_{j}}+\frac{\partial w_{j}}{\partial x_{i}}\right)\;;\;i,j=1,2,3$$

(88) $$\varepsilon_{ij,y}(w)=\frac{1}{2}\left(\frac{\partial w_{i}}{\partial y_{j}}+\frac{\partial w_{j}}{\partial y_{i}}\right)\;;\;i,j=1,2,3$$

The displacement field can be expressed by the series:

(89) $$u(x,y)=u^{o}(x)+u^{1}(x,y)\;\delta +u^{2}(x,y)\;\delta^{2}+....$$

$u^{o}(x)$ is a function on *x* (only). The terms $u^{1}(x,y)\;$, $u^{2}(x,y)$ are considered to be quasi-periodical. Using (87)–(89), it can be written as

(90) $$\varepsilon_{kh,x}(u)=\frac{1}{2}\left(\frac{\partial u_{k}}{\partial x_{h}}+\frac{\partial u_{h}}{\partial x_{k}}\right)\;\;=\frac{1}{2}\left(\frac{\partial u_{k}^{o}}{\partial x_{h}}+\frac{\partial u_{h}^{o}}{\partial x_{k}}\right)+\frac{\delta }{2}\left(\frac{\partial u_{k}^{1}}{\partial x_{h}}+\frac{\partial u_{h}^{1}}{\partial x_{k}}\right)+\frac{\delta }{2}\left(\frac{\partial u_{k}^{2}}{\partial y_{h}}+\frac{\partial u_{h}^{2}}{\partial y_{k}}\right)+....\;\;=\varepsilon_{kh,x}(u^{o})+\varepsilon_{kh,y}(u^{1})+\delta \left[\varepsilon_{kh,x}(u^{1})+\varepsilon_{kh,y}(u^{2})\right]+\delta^{2}\left[\ldots \right].+\ldots \;;\;k,h=1,2,3$$

or

(91) $$\varepsilon_{kh,x}(u)=\varepsilon_{kh}^{o}+\delta \;\varepsilon_{kh}^{1}+\ldots \;;\;k,h=1,2,3$$

where

(92) $$\varepsilon_{kh}^{o}=\varepsilon_{kh,x}(u^{o})+\varepsilon_{kh,y}(u^{1})\;k,h=1,2,3$$

(93) $$\varepsilon_{kh}^{1}=\left[\varepsilon_{kh,x}(u^{1})+\varepsilon_{kh,y}(u^{2})\right]\;;\;k,h=1,2,3$$

Applying the linear Hooke’s law results in

(94) $$\sigma_{ij}^{o}=C_{ijkh}\varepsilon_{kh}^{o}\;,\;i,j,k,h=1,2,3$$

From Equation (94), it follows that

(95) $$\frac{\partial \left(C_{ijkh}\varepsilon_{kh}^{o}\right)}{\partial y_{j}}=0\;,\;i,j,k,h=1,2,3$$

or

(96) $$\frac{\partial \left[C_{ijkh}\left(\varepsilon_{kh,x}^{}(u^{o})+\varepsilon_{kh,y}^{}(u^{1})\right)\right]}{\partial y_{j}}=0\;,\;i,j,k,h=1,2,3$$

The terms $\varepsilon_{kh,x}^{}(u^{o})$ depend only on *x.* Equation (96) can be written as

(97) $$-\frac{\partial \left[C_{ijkh}\;\varepsilon_{kh,y}^{}(u^{1})\right]}{\partial y_{j}}=\varepsilon_{kh,x}(u^{o})\frac{\partial C_{ijkh}}{\partial y_{j}}\;,\;i,j,k,h=1,2,3$$

introducing

(98) $$u^{1}=w^{kh}\varepsilon_{kh,x}^{}(u^{o})+k(x)$$

Using Equations (97) and (98) with *k*(*x*) an arbitrary function on *x*, we can obtain

(99) $$\varepsilon_{lm,y}^{}(u^{1})=\varepsilon_{kh,x}^{}(u^{o})\frac{1}{2}\left(\frac{\partial w_{l}^{kh}}{\partial y_{m}}+\frac{\partial w_{m}^{kh}}{\partial y_{l}}\right)=\varepsilon_{kh,x}^{}(u^{o})\varepsilon_{lm,y}^{}(w^{kh})$$

Equation (99) becomes

(100) $$-\varepsilon_{kh,x}^{}(u^{o})\frac{\partial \left[C_{ijlm}\varepsilon_{lm,y}(w^{kh})\right]}{\partial y_{j}}=\varepsilon_{kh,x}(u^{o})\frac{\partial C_{ijkh}}{\partial y_{j}}\;,\;i,j,k,h=1,2,3\;=1$$

Equation (100) is valid for any strain field $\varepsilon_{kh,x}(u^{o})$, so Equation (100) becomes

(101) $$-\frac{\partial \left[C_{ijlm}\varepsilon_{lm,y}(w^{kh})\right]}{\partial y_{j}}=\frac{\partial C_{ijkh}}{\partial y_{j}}\;,\;i,j,k,h=1,2,3$$

Using Green’s theorem, we obtain

(102) $$\begin{array}{l} \int_{\Gamma }\frac{\partial \left[C_{ijlm}\varepsilon_{lm,y}(w^{kh})\right]}{\partial y_{j}}v_{i}dV+\int_{\Gamma }C_{ijlm}\varepsilon_{lm,y}(w^{kh})\frac{\partial v_{i}}{\partial y_{j}}dV\\ =\int_{\partial \;\Gamma }u_{i}C_{ijlm}\varepsilon_{lm,y}(w^{kh})v_{i}^{}dS=0\;,\;i,j,k,h=1,2,3 \end{array}$$

(103) $$\begin{array}{l} \int_{\Gamma }\frac{\partial \left[C_{ijlm}\varepsilon_{lm,y}(w^{kh})\right]}{\partial y_{i}}v_{j}dV+\int_{\Gamma }C_{ijlm}\varepsilon_{lm,y}(w^{kh})\frac{\partial v_{j}}{\partial y_{i}}dV=\\ =\int_{\partial \;\Gamma }u_{j}C_{ijlm}\varepsilon_{lm,y}(w^{kh})v_{j}dS=0\;,\;i,j,k,h=1,2,3 \end{array}$$

In Equation (103), the indices *i* and *j* have been interchanged and the property $C_{ijlm}=C_{jilm}$ has been considered. From (102) and (103), it follows that

(104) $$\int_{\Gamma }\frac{\partial \left[C_{ijlm}\varepsilon_{lm,y}(w^{kh})\right]}{\partial y_{j}}v_{i}dV+\int_{\Gamma }\frac{\partial \left[C_{ijlm}\varepsilon_{lm,y}(w^{kh})\right]}{\partial y_{i}}v_{j}dV\;=2\int_{\Gamma }C_{ijlm}\varepsilon_{lm,y}(w^{kh})\frac{1}{2}\left(\frac{\partial v_{i}}{\partial y_{j}}+\frac{\partial v_{j}}{\partial y_{i}}\right)dV=2\int_{\Gamma }C_{ijlm}\varepsilon_{ij,y}(v)\varepsilon_{lm,y}(w^{kh})dV,\;i,j,k,h=1,2,3$$

Because $C_{ijlm}=C_{jilm}$, multiplying Equation (104) by v results in

(105) $$\frac{\partial \left[C_{ijlm}\varepsilon_{lm,y}(w^{kh})\right]}{\partial y_{j}}v_{i}=\frac{\partial C_{ijkh}}{\partial y_{j}}v_{i}$$

Interchanging the indices *i* and *j* results in

(106) $$\frac{\partial \left[C_{ijlm}\varepsilon_{lm,y}(w^{kh})\right]}{\partial y_{i}}v_{j}=\frac{\partial C_{ijkh}}{\partial y_{i}}v_{j}$$

The integration and addition of Equations (105) and (106) using Equation (104) offer

(107) $$\int_{\Gamma }C_{ijlm}\varepsilon_{ij,y}(v)\varepsilon_{lm,y}(w^{kh})dV=\int_{\Gamma }\frac{\partial C_{ijkh}}{\partial y_{j}}v_{i}dV$$

We must then find $w^{kh}$ in $V_{y}$ such that $\forall v\in V_{y}$, which verifies Equation (107). If $w^{kh}$ is obtained, then

(108) $$\sigma_{ij}^{o}=C_{ijkh}\left[\varepsilon_{kh,x}^{}(u^{o})+\varepsilon_{kh,y}^{}(u^{1})\right]\;=C_{ijkh}\left[\varepsilon_{kh,x}^{}(u^{o})+\varepsilon_{kh,x}^{}(u^{o})\;\varepsilon_{lm,y}\left(w^{kh}\right)\right]$$

By applying the average operator, it results in

(109) $$\langle \sigma_{ij}^{o}\rangle =\langle C_{ijkh}\varepsilon_{kh,x}(u^{o})\rangle +\langle C_{ijkh}\varepsilon_{kh,x}(u^{o})\varepsilon_{lm,y}(u^{kh})\rangle \;=\langle C_{ijkh}\rangle \varepsilon_{kh,x}(u^{o})+\langle C_{ijkh}\varepsilon_{lm,y}(u^{kh})\rangle \varepsilon_{kh,x}(u^{o})$$

This produces

(110) $$\langle \sigma_{ij}^{o}\rangle =\left[\langle C_{ijkh}\rangle +\langle C_{ijkh}\varepsilon_{lm,y}(u^{kh})\rangle \right]\varepsilon_{kh,x}(u^{o})$$

A comparison of Equation (110) can be made with

(111) $$\langle \sigma_{ij}^{o}\rangle =C_{ijkh}^{o}{\tilde{\varepsilon }}_{kh}(u^{o})$$

and, if we denote $\varepsilon_{kh,x}(u^{o})\equiv {\tilde{\varepsilon }}_{kh}(u^{o})$, the homogenized coefficients can be obtained:

(112) $$C_{ijkh}^{o}=\langle C_{ijkh}\rangle +\langle C_{ijkh}\varepsilon_{lm,y}(u^{kh})\rangle$$

Therefore, there are two ways to obtain the homogenized coefficients:

- Using the local equations, the strain and stress field and the averages are determined, obtaining the homogenized coefficients;
- Using the variational formulation and determining the function $w^{kh}$ can also help us to determine the homogenized coefficients.

For the fiber-reinforced composite, there is a class of solutions $w^{kh}$, with *k*,*h* = 1,2,3 satisfying

(113) $$-\frac{\partial \left[C_{ijlm}\varepsilon_{lm,y}\left(w\right)\right]}{\partial y_{j}}=\frac{\partial C_{ijkh}}{\partial y_{j}}\;,\;i=1,2,3$$

with the boundary conditions

(114) $${w^{kh}|}_{\partial \Gamma }=0$$

and

(115) $$\langle w^{kh}\rangle =0$$

If $\left(x_{1},x_{2},x_{3}\right)$ are the principal material axes, we state

(116) $$C_{1111}=C_{11};\;C_{2222}=C_{22};\;C_{1122}=C_{1133}=C_{12};\;C_{2211}=C_{3311}=C_{21}\;C_{3322}=C_{2233}=C_{23};\;C_{3333}=C_{33};\;C_{4444}=\left(C_{22}-C_{23}\right)/2;\;C_{5555}=C_{44};\;\;C_{6666}=C_{44}$$

The other components of $C_{ijkl}$ are zero (we work with a transversely isotropic material). The stress–strain relation becomes

(117) $$\left\{\begin{array}{c} \sigma_{22}\\ \sigma_{33}\\ \tau_{23} \end{array}\right\}=\left[\begin{array}{ccc} C_{22} & C_{23} & 0\\ C_{23} & C_{33} & 0\\ 0 & 0 & \frac{C_{22}-C_{23}}{2} \end{array}\right]\left\{\begin{array}{c} \varepsilon_{22}\\ \varepsilon_{33}\\ \gamma_{23} \end{array}\right\}$$

or

(118) $$\left\{\sigma \right\}=\left[C\right]{\left\{\varepsilon \right\}}^{o}$$

The equilibrium conditions in Equation (71) are

(119) $$\left[\begin{array}{ccc} \frac{\partial }{\partial y_{2}} & 0 & \frac{\partial }{\partial y_{3}}\\ 0 & \frac{\partial }{\partial y_{3}} & \frac{\partial }{\partial y_{2}} \end{array}\right]\left\{\begin{array}{c} \sigma_{22}\\ \sigma_{33}\\ \tau_{23} \end{array}\right\}=0$$

Equation (119) can be expressed in compact form:

(120) $$\left[\partial \right]\left\{\sigma \right\}=\left[\partial \right]\left[C\right]{\left\{\varepsilon \right\}}^{o}=0$$

Equation (92) becomes

(121) $${\left\{\varepsilon \right\}}^{o}={\left\{\varepsilon \right\}}_{,x}^{u^{o}}+{\left\{\varepsilon \right\}}_{,y}^{u^{1}}$$

and (120) becomes

(122) $$-\left[\begin{array}{ccc} \frac{\partial }{\partial y_{2}} & 0 & \frac{\partial }{\partial y_{3}}\\ 0 & \frac{\partial }{\partial y_{3}} & \frac{\partial }{\partial y_{2}} \end{array}\right]\left[\begin{array}{ccc} C_{22}^{\left(\lambda \right)} & C_{23}^{\left(\lambda \right)} & 0\\ C_{23}^{\left(\lambda \right)} & C_{33}^{\left(\lambda \right)} & 0\\ 0 & 0 & \frac{C_{22}^{\left(\lambda \right)}-C_{23}^{\left(\lambda \right)}}{2} \end{array}\right]{\left\{\varepsilon \right\}}_{,y}^{u^{1}}\;=\left[\begin{array}{ccc} \frac{\partial }{\partial y_{2}} & 0 & \frac{\partial }{\partial y_{3}}\\ 0 & \frac{\partial }{\partial y_{3}} & \frac{\partial }{\partial y_{2}} \end{array}\right]\left[\begin{array}{ccc} C_{22}^{\left(\lambda \right)} & C_{23}^{\left(\lambda \right)} & 0\\ C_{23}^{\left(\lambda \right)} & C_{33}^{\left(\lambda \right)} & 0\\ 0 & 0 & \frac{C_{22}^{\left(\lambda \right)}-C_{23}^{\left(\lambda \right)}}{2} \end{array}\right]{\left\{\varepsilon \right\}}_{,x}^{u^{o}}$$

Considering the plane strain loading conditions, we obtain

(123) $$\left[\begin{array}{ccc} \frac{\partial }{\partial y_{2}} & 0 & \frac{\partial }{\partial y_{3}}\\ 0 & \frac{\partial }{\partial y_{3}} & \frac{\partial }{\partial y_{2}} \end{array}\right]\left[\begin{array}{ccc} C_{22}^{\left(\lambda \right)} & C_{23}^{\left(\lambda \right)} & 0\\ C_{23}^{\left(\lambda \right)} & C_{33}^{\left(\lambda \right)} & 0\\ 0 & 0 & \frac{C_{22}^{\left(\lambda \right)}-C_{23}^{\left(\lambda \right)}}{2} \end{array}\right]{\left\{\varepsilon \right\}}_{,y}^{u^{1}}=0$$

Using the determined functions $w^{kh}$, it can be deduced that

(124) $${\left\{\varepsilon \right\}}_{,y}^{u^{1}}=\left[\begin{array}{ccc} \varepsilon_{22}\left(w^{22}\right) & \varepsilon_{22}\left(w^{33}\right) & \varepsilon_{22}\left(w^{23}\right)\\ \varepsilon_{33}\left(w^{22}\right) & \varepsilon_{33}\left(w^{33}\right) & \varepsilon_{33}\left(w^{23}\right)\\ \varepsilon_{23}\left(w^{22}\right) & \varepsilon_{23}\left(w^{33}\right) & \varepsilon_{23}\left(w^{23}\right) \end{array}\right]{\left\{\varepsilon \right\}}_{,x}^{u^{o}}$$

or, in an alternative form,

(125) $${\left\{\varepsilon \right\}}_{,y}^{u^{1}}=\left[\begin{array}{ccc} \left\{\varepsilon \left(w^{22}\right)\right\} & \left\{\varepsilon \left(w^{33}\right)\right\} & \left\{\varepsilon \left(w^{23}\right)\right\} \end{array}\right]{\left\{\varepsilon \right\}}_{,x}^{u^{o}}$$

Additionally,

(126) $$\left[\begin{array}{ccc} \frac{\partial }{\partial y_{2}} & 0 & \frac{\partial }{\partial y_{3}}\\ 0 & \frac{\partial }{\partial y_{3}} & \frac{\partial }{\partial y_{2}} \end{array}\right]\left[\begin{array}{ccc} C_{22}^{\left(\lambda \right)} & C_{23}^{\left(\lambda \right)} & 0\\ C_{23}^{\left(\lambda \right)} & C_{33}^{\left(\lambda \right)} & 0\\ 0 & 0 & \frac{C_{22}^{\left(\lambda \right)}-C_{23}^{\left(\lambda \right)}}{2} \end{array}\right]\left[\begin{array}{ccc} \left\{\varepsilon \left(w^{22}\right)\right\} & \left\{\varepsilon \left(w^{33}\right)\right\} & \left\{\varepsilon \left(w^{23}\right)\right\} \end{array}\right]{\left\{\varepsilon \right\}}_{,x}^{u^{o}}=0$$

(127) $$\left[\begin{array}{ccc} \left\{\varepsilon \left(w^{22}\right)\right\} & \left\{\varepsilon \left(w^{33}\right)\right\} & \left\{\varepsilon \left(w^{23}\right)\right\} \end{array}\right]{\left\{\varepsilon \right\}}_{,x}^{u^{o}}=0$$

Equation (127) should remain valid for all ${\left\{\varepsilon \right\}}_{,x}^{u^{o}}$. Equation (113) becomes

(128) $$-\frac{C_{ijlm}^{\left(\lambda \right)}\partial \varepsilon_{lm,y}^{\left(\lambda \right)}(w^{kh})}{\partial y_{j}}=0\;,\;i=1,2,3$$

In we consider the case of plane strain i=2,3 and j=2,3,

(129) $$C_{22}^{\left(\lambda \right)}\frac{\partial \varepsilon_{22}\left(w^{kh}\right)}{\partial y_{2}}+C_{23}^{\left(\lambda \right)}\frac{\partial \varepsilon_{33}\left(w^{kh}\right)}{\partial y_{2}}+\frac{1}{2}\left[C_{22}^{\left(\lambda \right)}-C_{23}^{\left(\lambda \right)}\right]\frac{\partial \varepsilon_{23}\left(w^{kh}\right)}{\partial y_{3}}=0$$

and

(130) $$C_{23}^{\left(\lambda \right)}\frac{\partial \varepsilon_{22}\left(w^{kh}\right)}{\partial y_{3}}+C_{22}^{\left(\lambda \right)}\frac{\partial \varepsilon_{33}\left(w^{kh}\right)}{\partial y_{3}}+\frac{1}{2}\left[C_{22}^{\left(\lambda \right)}-C_{23}^{\left(\lambda \right)}\right]\frac{\partial \varepsilon_{23}\left(w^{kh}\right)}{\partial y_{2}}=0$$

The solution is

(131) $$w^{kh}=\{\begin{array}{c} w^{kh,(f)}\;for\;y\in V_{f}\\ w^{kh,(m)}\;for\;y\in V_{m} \end{array}$$

satisfying the boundary conditions

(132) $${w^{kh,(f)}|}_{\partial \Gamma }=\;{w^{kh,(m)}|}_{\partial \Gamma };\;\sigma_{ij}^{\left(f\right)}n_{j}=\sigma_{ij}^{\left(m\right)}n_{j}$$

The boundary conditions for the RUC are: $u_{i}^{}=\alpha_{ij}y_{j}$. It is possible to show that the average strain is: $\langle \varepsilon_{ij}\rangle ={\bar{\varepsilon }}_{ij}=\alpha_{ij}$. Let us denote the displacement field by $w^{*}$ having the property ${w^{*}|}_{\partial \Gamma }={u\;|}_{\partial \Gamma }$ and ${\bar{\varepsilon }}_{kh}\left(w^{*}\right)=\alpha_{ij}$. Due to the existing symmetry in the distribution of the unit cell it can be concluded that $\langle w^{*}\rangle =0$. The field w is introduced as

(133) $$w=w^{*}-u$$

with the boundary conditions

(134) $${w\;|}_{\partial \Gamma }=0$$

(135) $$\langle w\rangle =\langle w^{*}-u\rangle =\langle w^{*}\rangle -\langle u\rangle =0$$

This function (w) verifies the condition of zero average and value zero on the contour, and it verifies Equation (130). For the “quasi-periodical fields” $u{\;}_{1}$, it follows that

(136) $$u_{1}=\alpha_{22}\left\{\begin{array}{c} \frac{w_{22}^{*}}{\alpha_{22}}-y_{2}\\ 0 \end{array}\right\}+\alpha_{33}\left\{\begin{array}{c} 0\\ \frac{w_{22}^{*}}{\alpha_{22}}-y_{2} \end{array}\right\}$$

The strain field is

(137) $$\varepsilon_{22}\left(w^{22}\right)=\frac{\varepsilon_{22}\left(w^{*}\right)}{\alpha_{22}}-1\;;\;\varepsilon_{33}\left(w^{22}\right)=0$$

(138) $$\varepsilon_{22}\left(w^{33}\right)=\frac{\varepsilon_{22}\left(w^{*}\right)}{\alpha_{33}}-1\;;\;\varepsilon_{33}\left(w^{33}\right)=0$$

and

(139) $$\varepsilon_{22}\left(u_{1}\right)=\varepsilon_{22}\left(w^{*}\right)-\alpha_{22}\;;\varepsilon_{33}\left(u_{1}\right)=\varepsilon_{33}\left(w^{*}\right)-\alpha_{33}\;$$

or

(140) $${\bar{\varepsilon }}_{22}\left(w^{22}\right)=\frac{{\bar{\varepsilon }}_{22}\left(w^{*}\right)}{\alpha_{22}}-1=0\;;\;{\bar{\varepsilon }}_{33}\left(w^{33}\right)=\frac{{\bar{\varepsilon }}_{33}\left(w^{*}\right)}{\alpha_{33}}-1=0\;$$

For the fiber-reinforced unidirectional composite, the homogenized coefficients can be obtained with the following relations:

(141) $$C_{ijkh}^{o}=\langle C_{ijkh}\rangle +\langle C_{ijkh}\varepsilon_{lm,y}\left(w^{kh}\right)\rangle \;=\frac{1}{V}\int_{\Gamma }C_{ijkh}dV+\frac{1}{V}\int_{\Gamma }C_{ijkh}\varepsilon_{lm}\left(w^{*}\right)dV\;=\frac{1}{V}\left(C_{ijkh}^{\left(f\right)}V_{f}+C_{ijkh}^{\left(m\right)}V_{m}\right)+\frac{1}{V}\left(C_{ijlm}^{\left(f\right)}{\bar{\varepsilon }}_{lm}^{\left(f\right)}\left(w\right)V_{f}+C_{ijlm}^{\left(m\right)}{\bar{\varepsilon }}_{lm}^{\left(m\right)}\left(w\right)V_{m}\right)$$

where

(142) $$\alpha_{lm}{\bar{\varepsilon }}_{lm}^{\left(f\right)}\left(w\right)={\bar{\varepsilon }}_{lm}^{\left(f\right)}\left(w^{*}\right)-\alpha_{lm}\;;\;\alpha_{lm}{\bar{\varepsilon }}_{lm}^{\left(m\right)}\left(w\right)={\bar{\varepsilon }}_{lm}^{\left(m\right)}\left(w^{*}\right)-\alpha_{lm}$$

Thus, we have

(143) $$C_{ijkh}^{o}=v_{f}C_{ijkh}^{\left(f\right)}+v_{m}C_{ijkh}^{\left(m\right)}+v_{f}C_{ijkh}^{\left(f\right)}\left[\frac{{\bar{\varepsilon }}_{lm}^{\left(f\right)}\left(w*\right)}{\alpha_{lm}}-1\right]+v_{m}C_{ijkh}^{\left(m\right)}\left[\frac{{\bar{\varepsilon }}_{lm}^{\left(m\right)}\left(w*\right)}{\alpha_{lm}}-1\right]$$

As a result (considering the plane strain loading conditions),

(144) $$C_{22}^{o}=v_{f}C_{22}^{\left(f\right)}+v_{m}C_{22}^{\left(m\right)}+v_{f}C_{22}^{\left(f\right)}\left[\frac{{\bar{\varepsilon }}_{22}^{\left(f\right)}\left(w*\right)}{\alpha_{22}}-1\right]+v_{m}C_{22}^{\left(m\right)}\left[\frac{{\bar{\varepsilon }}_{22}^{\left(m\right)}\left(w*\right)}{\alpha_{22}}-1\right]\;=v_{f}C_{22}^{\left(f\right)}\frac{{\bar{\varepsilon }}_{22}^{\left(f\right)}\left(w*\right)}{\alpha_{22}}+v_{m}C_{22}^{\left(m\right)}\frac{{\bar{\varepsilon }}_{22}^{\left(m\right)}\left(w*\right)}{\alpha_{22}}$$

and

(145) $$C_{23}^{o}=v_{f}C_{23}^{\left(f\right)}+v_{m}C_{23}^{\left(m\right)}+v_{f}C_{23}^{\left(f\right)}\left[\frac{{\bar{\varepsilon }}_{33}^{\left(f\right)}\left(w*\right)}{\alpha_{33}}-1\right]+v_{m}C_{23}^{\left(m\right)}\left[\frac{{\bar{\varepsilon }}_{33}^{\left(m\right)}\left(w*\right)}{\alpha_{33}}-1\right]\;=v_{f}C_{23}^{\left(f\right)}\frac{{\bar{\varepsilon }}_{33}^{\left(f\right)}\left(w*\right)}{\alpha_{33}}+v_{m}C_{23}^{\left(m\right)}\frac{{\bar{\varepsilon }}_{33}^{\left(m\right)}\left(w*\right)}{\alpha_{33}}$$

## 4. The Mori–Tanaka Model

### 4.1. Mathematical Model

**Figure 5:**
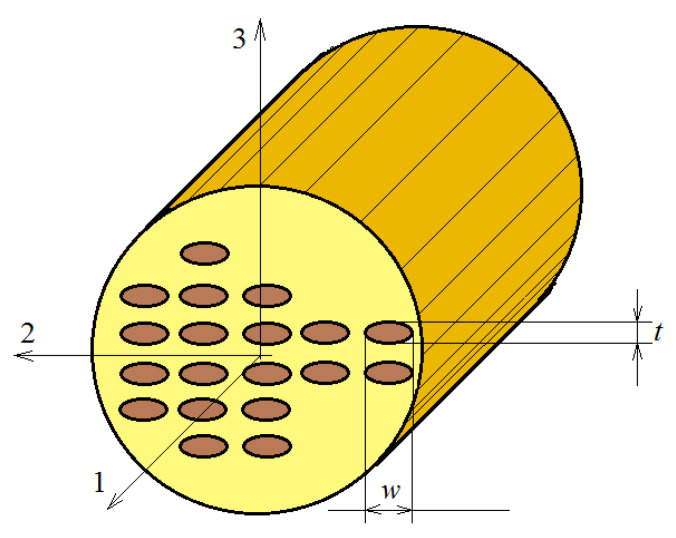
Reinforced composite.

In the following section, the mathematical model proposed by Mori and Tanaka is applied to obtain the engineering parameters that define Hooke’s law for a one-dimensional fiber-reinforced composite [34]. We consider an epoxy matrix with a visco-elastic response, reinforced with monotonous and parallel aligned carbon fibers that are uniformly distributed inside the resin (Figure 5). The resulting material has an orthotropic behavior. However, there are applications where the fibers are elliptical cylinders. These cylinders are randomly distributed, and the behavior of the material is a transverse isotropic.

The theory developed in [78] is applied in [28] for a reinforced material with continuous cylindrical fibers with an elliptic section. To solve this problem, Mori–Tanaka’s [34] mean-field theory is used. In [79,80], the two phases of the composite are two isotropic materials.

We consider a comparison material (CM). In the CM, there is a linear relation between the mean strain field ε° and the mean stress field $\bar{\sigma }$:

(146) $$\bar{\sigma }=C_{m}\varepsilon^{0}$$

The average strain field in the RUC is $\varepsilon^{m}=\varepsilon^{0}+\bar{\varepsilon }$ and the mean stress field is $\sigma^{m}=\bar{\sigma }+\tilde{\sigma }$. This results in

(147) $$\sigma^{m}=\bar{\sigma }+\tilde{\sigma }=C_{m}(\varepsilon^{0}+\bar{\varepsilon })$$

The mean strain fields in the fiber and in the matrix are differentiated through an additional term $\varepsilon^{pt}$, hence $\varepsilon^{f}=\varepsilon^{m}+\varepsilon^{pt}=\varepsilon^{0}+\tilde{\varepsilon }+\varepsilon^{pt}$. In a similar way, the average stress field differs by the term $\sigma^{pt}$ and, therefore, $\sigma^{f}=\bar{\sigma }+\tilde{\sigma }+\sigma^{pt}$. The generalized Hooke law becomes

(148) $$\sigma^{f}=\bar{\sigma }+\tilde{\sigma }+\sigma^{pt}=C_{f}(\varepsilon^{0}+\tilde{\varepsilon }+\varepsilon^{pt})$$

or

(149) $$\sigma^{f}=\bar{\sigma }+\tilde{\sigma }+\sigma^{pt}=C_{f}(\varepsilon^{0}+\tilde{\varepsilon }+\varepsilon^{pt})=C_{m}(\varepsilon^{0}+\tilde{\varepsilon }+\varepsilon^{pt}-\varepsilon^{*})$$

We introduce $\varepsilon^{pt}$ in Equation (149).

(150) $$\varepsilon^{pt}=P\varepsilon^{*}$$

The Eshelby transformation tensor *P* from Equation (150) is presented in Appendix A (where *P_ikjl_ = P_jikl_ = P_ijtk_*). The average stress in the whole RUC is

(151) $$\begin{array}{l} \bar{\sigma }=v_{f}\sigma_{f}+v_{m}\sigma_{m}=v_{f}(\bar{\sigma }+\tilde{\sigma }+\sigma^{pt})+v_{m}(\bar{\sigma }+\tilde{\sigma })\\ =(v_{f}+v_{m})\bar{\sigma }+(v_{f}+v_{m})\tilde{\sigma }+v_{f}\sigma^{pt}=\bar{\sigma }+\tilde{\sigma }+v_{f}\sigma^{pt} \end{array}$$

which reduces to

(152) $$\tilde{\sigma }=-v_{f}\sigma^{pt}$$

In a similar way, we can obtain

(153) $$\bar{\varepsilon }=-v_{f}(\varepsilon^{pt}-\varepsilon^{*})=-v_{f}(P\varepsilon^{*}-\varepsilon^{*})=-v_{f}(P-I)\varepsilon^{*}$$

*I* denotes the unit tensor. Equations (147) and (149) yield

(154) $$C_{f}\left[\varepsilon^{0}-v_{f}\left(P-I\right)\varepsilon^{*}+P\varepsilon^{*}\right]=C_{m}\left[\varepsilon^{0}-v_{f}\left(P-I\right)\varepsilon^{*}+P\varepsilon^{*}-\varepsilon^{*}\right]$$

or

(155) $$\left[C_{f}\left(-v_{f}\left(P-I\right)+P\right)+C_{m}\left(v_{f}\left(P-I\right)-P+I\right)\right]\varepsilon^{*}+\left(C_{f}-C_{m}\right)\varepsilon^{0}=0$$

or

(156) $$\left[C_{f}\left(v_{m}P+v_{f}I\right)-C_{m}v_{m}(P-I)\right]\varepsilon^{*}+\left(C_{f}-C_{m}\right)\varepsilon^{0}=0$$

and

(157) $$\left[v_{m}\left(C_{f}-C_{m}\right)P+v_{f}\left(C_{f}-C_{m}\right)+C_{m}\right]\varepsilon^{*}+\left(C_{f}-C_{m}\right)\varepsilon^{0}=0$$

The final form is

(158) $$\left[\left(C_{f}-C_{m}\right)\left(v_{m}P+v_{f}I\right)+C_{m}\right]\varepsilon^{*}+\left(C_{f}-C_{m}\right)\varepsilon^{0}=0$$

This offers

(159) $$\begin{array}{l} \varepsilon_{11}^{*}=\frac{1}{A}\left(A_{11}\varepsilon_{11}^{o}+A_{12}\varepsilon_{22}^{o}+A_{13}\varepsilon_{33}^{o}\right)\;;\;\\ \varepsilon_{11}^{*}=\frac{1}{A}\left(A_{21}\varepsilon_{11}^{o}+A_{22}\varepsilon_{22}^{o}+A_{23}\varepsilon_{33}^{o}\right)\;;\\ \varepsilon_{11}^{*}=\frac{1}{A}\left(A_{31}\varepsilon_{11}^{o}+A_{32}\varepsilon_{22}^{o}+A_{33}\varepsilon_{33}^{o}\right)\;. \end{array}$$

The coefficients $A_{ij}$ are presented in Appendix A [30]. The shear strain is [30]

(160) $$\varepsilon_{12}^{*}=\frac{\left(G_{12,f}-G_{m}\right)}{\left(G_{12,f}-G_{m}\right)\left(2v_{m}P_{1212}+v_{f}\right)+G_{m}}\varepsilon_{12}^{0}$$

(161) $$\varepsilon_{23}^{*}=\frac{\left(G_{23,f}-G_{m}\right)}{\left(G_{23,f}-G_{m}\right)\left(2v_{m}P_{2323}+v_{f}\right)+G_{m}}\varepsilon_{23}^{0}$$

(162) $$\varepsilon_{31}^{*}=\frac{\left(G_{31,f}-G_{m}\right)}{\left(G_{31,f}-G_{m}\right)\left(2v_{m}P_{3131}+v_{f}\right)+G_{m}}\varepsilon_{31}^{0}$$

Equations (158)–(162) can now be used to determine the elastic/viscoelastic parameters of a composite, which is considered as an orthotropic body. To compute the Young’s modulus $E_{m}$, the composite specimen is subjected to a pure traction ${\bar{\sigma }}_{11}$. This results in the following equation: ${\bar{\sigma }}_{11}=E_{11}{\bar{\varepsilon }}_{11}$ and ${\bar{\sigma }}_{11}=E_{m}{\bar{\varepsilon }}_{11}^{0}$; ${\bar{\varepsilon }}_{22}^{0}={\bar{\varepsilon }}_{33}^{0}=-\nu_{m}{\bar{\varepsilon }}_{11}^{0}$.

Equation (158) produces

(163) $$\begin{array}{ll} {\bar{\varepsilon }}_{11}={\bar{\varepsilon }}_{11}^{0}+ & v_{f}{\bar{\varepsilon }}_{11}^{*}={\bar{\varepsilon }}_{11}^{0}+v_{f}\left(\frac{A_{11}}{A}{\bar{\varepsilon }}_{11}^{0}+\frac{A_{12}}{A}{\bar{\varepsilon }}_{22}^{0}+\frac{A_{13}}{A}{\bar{\varepsilon }}_{33}^{0}\right)\\ & \;={\bar{\varepsilon }}_{11}^{0}\left(1+v_{f}a_{11}\right)-v_{f}a_{12}v_{m}{\bar{\varepsilon }}_{11}^{0}-v_{f}a_{13}v_{m}{\bar{\varepsilon }}_{11}^{0}\\ & \;={\bar{\varepsilon }}_{11}^{0}\left[1+v_{f}\left[a_{11}-v_{m}\left(a_{12}+a_{13}\right)\right]\right] \end{array}$$

Here, we show that $a_{ij}^{}=A_{ij}^{}/A$, $A_{ij}^{}$, and A is presented in Appendix A; see rel. (A6). This results in

(164) $$E_{11}=\frac{{\bar{\varepsilon }}_{11}^{0}}{{\bar{\varepsilon }}_{11}}E_{m}=\frac{E_{m}}{1+v_{f}\left[a_{11}-v_{m}\left(a_{12}+a_{13}\right)\right]}$$

For the other directions, in a similar way, we obtain the following equations:

(165) $$E_{22}=\frac{{\bar{\varepsilon }}_{22}^{0}}{{\bar{\varepsilon }}_{22}}E_{m}=\frac{E_{m}}{1+v_{f}\left[a_{22}-v_{m}\left(a_{21}+a_{23}\right)\right]}$$

and

(166) $$E_{33}=\frac{{\bar{\varepsilon }}_{33}^{0}}{{\bar{\varepsilon }}_{33}}E_{m}=\frac{E_{m}}{1+v_{f}\left[a_{33}-v_{m}\left(a_{31}+a_{32}\right)\right]}$$

Considering the shear moduli, we have

(167) $${\bar{\sigma }}_{12}=2G_{12}{\bar{\varepsilon }}_{12\;}\;;\;{\bar{\sigma }}_{12}=2G_{m}{\bar{\varepsilon }}_{12}^{0}\;$$

However,

(168) $${\bar{\varepsilon }}_{12}={\bar{\varepsilon }}_{12}^{0}+v_{f}{\bar{\varepsilon }}_{12}^{*}=\varepsilon_{12}^{*}-v_{f}\frac{G_{12,f}-G_{m}}{\left(G_{12,f}-G_{m}\right)\left(2v_{m}P_{1212}+v_{f}\right)+G_{m}}\varepsilon_{12}^{0}$$

Using Equations (167) and (168) produces $G_{12}$:

(169) $$G_{12}=G_{m}\left(1+\frac{v_{f}}{\frac{G_{m}}{G_{12,f}-G_{m}}+2v_{m}P_{1212}}\right)$$

In the same way, we obtain

(170) $$G_{23}=G_{m}\left(1+\frac{v_{f}}{\frac{G_{m}}{G_{23,f}-G_{m}}+2v_{m}P_{2323}}\right)$$

and

(171) $$G_{31}=G_{m}\left(1+\frac{v_{f}}{\frac{G_{m}}{G_{31,f}-G_{m}}+2v_{m}P_{3131}}\right)$$

The Poisson ratio is computed using the formulas

(172) $${\bar{\varepsilon }}_{22}=-v_{m}{\bar{\varepsilon }}_{11}\;;\;{\bar{\varepsilon }}_{22}^{0}=\;{\bar{\varepsilon }}_{33}^{0}=-v_{m}{\bar{\varepsilon }}_{11}^{0}\;$$

Note that

(173) $$\begin{array}{l} {\bar{\varepsilon }}_{11}={\bar{\varepsilon }}_{11}^{0}+v_{f}{\bar{\varepsilon }}_{11}^{*}={\bar{\varepsilon }}_{11}^{0}+v_{f}a_{11}{\bar{\varepsilon }}_{11}^{0}+v_{f}a_{12}{\bar{\varepsilon }}_{22}^{0}+v_{f}a_{13}{\bar{\varepsilon }}_{33}^{0}=\\ ={\bar{\varepsilon }}_{11}^{0}\left(1+v_{f}a_{11}\right)+v_{f}a_{12}{\bar{\varepsilon }}_{22}^{0}+v_{f}a_{13}{\bar{\varepsilon }}_{33}^{0} \end{array}$$

and

(174) $${\bar{\varepsilon }}_{22}={\bar{\varepsilon }}_{22}^{0}+v_{f}{\bar{\varepsilon }}_{22}^{*}=v_{f}a_{21}{\bar{\varepsilon }}_{11}^{0}+{\bar{\varepsilon }}_{22}^{0}\left(1+v_{f}a_{22}\right)+v_{f}a_{23}{\bar{\varepsilon }}_{33}^{0}$$

or

(175) $${\bar{\varepsilon }}_{11}=\left[\left(1+v_{f}a_{11}\right)-v_{f}a_{12}v_{m}-v_{f}a_{13}v_{m}\right]{\bar{\varepsilon }}_{11}^{0}=\left[v_{f}a_{21}-v_{m}\left(1+v_{f}a_{22}\right)-v_{m}v_{f}a_{23}\right]{\bar{\varepsilon }}_{11}^{0}$$

Introducing Equation (174) into Equation (175) produces

(176) $$v_{12}=-\frac{{\bar{\varepsilon }}_{22}}{{\bar{\varepsilon }}_{11}}=-\frac{v_{f}a_{21}-v_{m}\left(1+v_{f}a_{22}\right)-v_{m}v_{f}a_{23}}{1+v_{f}a_{11}-v_{f}a_{12}v_{m}-v_{f}a_{13}v_{m}}$$

which can be written as

(177) $$v_{12}=\frac{v_{m}-v_{f}\left[a_{22}-v_{m}\left(a_{21}+a_{23}\right)\right]}{1+v_{f}\left[a_{11}-v_{m}\left(a_{12}+a_{13}\right)\right]}$$

In the same way, it produces

(178) $$v_{23}=\frac{v_{m}-v_{f}\left[a_{22}-v_{m}\left(a_{21}+a_{23}\right)\right]}{1+v_{f}\left[a_{33}-v_{m}\left(a_{7}+a_{8}\right)\right]}$$

and

(179) $$v_{31}=\frac{v_{m}-v_{f}\left[a_{33}-v_{m}\left(a_{31}+a_{32}\right)\right]}{1+v_{f}\left[a_{11}-v_{m}\left(a_{12}+a_{13}\right)\right]}$$

## 5. The Finite Element Method Used to Obtain the Creep Response

**Figure 6:**
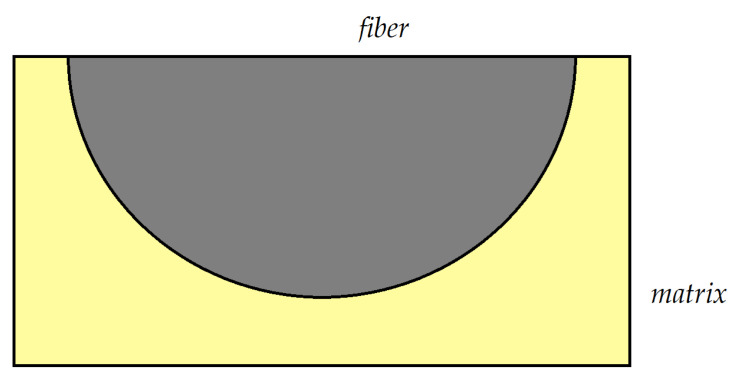
Finite element model 1 for a RUC.

**Figure 7:**
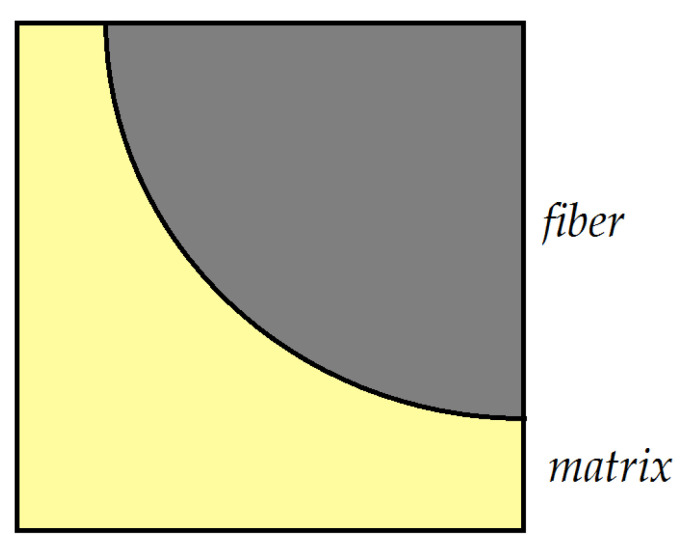
Finite element model 2 for a RUC.

Recently, FEM has become the main method used for the study of elastic systems, as it is able to address a multitude of situations and types of materials, including composite materials [81]. Specialized problems are also studied, such as the influence of temperature on the stresses that appear in the analyzed structures [82]. In [83], a model is presented for the study of a composite reinforced with silicon carbide fibers. A similar model is addressed in [84]. Bodies with transverse isotropy were also studied, as in [11,85]. If we are dealing with microstructured systems, where a unit cell can be identified, the geometric symmetry allows the analysis to be conducted only on a quarter or half of the unit cell, on a unit previously defined as the “representative unit cell” (RUC). The unit cell model with finite elements is presented in Figure 6 and Figure 7; two models of a RUC that are used in various applications are also presented (Models 1 and 2).

The mechanical constants used in the application are

*E_m_ =* 4.14 GPa *; v_m_ =* 0.22; *E_f_ =* 86.90 GPa; *v_f_* = 0.34 (180)

The results of the analysis are shown in Table 1, Table 2, Table 3, Table 4, Table 5 and Table 6 (in these tables, $\bar{\sigma }$ is the average stress and $\bar{\varepsilon }$ is the average strain).

In this paper, we used a three-dimensional model to obtain the shear modulus and Poisson’s ratios in a plane perpendicular to $x_{2}x_{3}$.

A few of the foregoing models are listed in Table 7, for which the results using finite element analysis are obtained.

There are some discrepancies between the present FE results and those presented in [49]. With respect to these discrepancies, the following verification should be considered. If the boundary condition for the FE model is taken as $u_{i}=\alpha_{ij}x_{j}$ (where $\alpha_{ij}=\alpha_{ji}$), the average strain should be equal to ${\bar{\varepsilon }}_{ij}=\alpha_{ij}$**.** This can be demonstrated as follows:

(181) $${\bar{\varepsilon }}_{ij}=\frac{1}{V}\int_{\Gamma }\varepsilon_{ij}dV=\frac{1}{2V}\int_{\Gamma }\left(\frac{\partial u_{j}}{\partial x_{i}}+\frac{\partial u_{i}}{\partial x_{j}}\right)dV$$

By applying Green’s theorem, it follows that

(182) $${\bar{\varepsilon }}_{ij}=\frac{1}{2V}\int_{\partial \;\Gamma }\left(n_{i}u_{j}+n_{j}u_{i}\right)ds=\;=\frac{1}{2V}\left(\int_{\partial \;\Gamma }n_{i}\alpha_{jk}x_{k}ds+\int_{\partial \;\Gamma }n_{j}\alpha_{il}x_{l}ds\right)=\;=\frac{1}{2V}\left(\alpha_{jk}\int_{\partial \;\Gamma }n_{i}x_{k}ds+\alpha_{il}\int_{\partial \;\Gamma }n_{j}x_{l}ds\right)$$

or

(183) $${\bar{\varepsilon }}_{ij}=\frac{1}{2V}\left(\alpha_{jk}\int_{\;\Gamma }\frac{\partial x_{k}}{\partial x_{i}}dV+\alpha_{il}\int_{\;\Gamma }\frac{\partial x_{l}}{\partial x_{j}}dV\right)=\;=\frac{1}{2V}\left(\alpha_{jk}\int_{\;\Gamma }\delta_{ki}dV+\alpha_{il}\int_{\;\Gamma }\delta_{lj}dV\right)=\frac{1}{2V}\left(\alpha_{ji}+\alpha_{ij}\right)=\alpha_{ij}$$

The discrepancy identified with the results of [49] can be attributed to the different type of finite elements used.

Therefore, we obtain average strains and stresses, viz., ${\bar{\sigma }}_{22},{\bar{\sigma }}_{33},{\bar{\sigma }}_{11},{\bar{\sigma }}_{23}={\bar{\tau }}_{23},{\bar{\varepsilon }}_{22},$ ${\bar{\varepsilon }}_{33},{\bar{\varepsilon }}_{11},{\bar{\varepsilon }}_{23}=1/2\;\gamma_{23}$. Using these values, it is now possible to obtain the mechanical constants of the studied composite [56]. To determine the longitudinal elastic modulus $E_{11}$, we use the well-known rule of mixture:

(184) $$E_{11}=E_{f}\nu_{f}+E_{m}\nu_{m}$$

where

(185) $$\nu_{f}=\frac{A_{f}}{A}\;;\;\nu_{f}=\frac{A_{m}}{A}\;$$

The following relations exist:

(186) $${\bar{\sigma }}_{22}=C_{22}{\bar{\varepsilon }}_{22}+C_{23}{\bar{\varepsilon }}_{33};\;{\bar{\sigma }}_{33}=C_{23}{\bar{\varepsilon }}_{22}+C_{22}{\bar{\varepsilon }}_{33};\;{\bar{\sigma }}_{11}=C_{12}\left({\bar{\varepsilon }}_{22}+{\bar{\varepsilon }}_{33}\right);\;{\bar{\tau }}_{23}=C_{66}\;{\bar{\gamma }}_{23}$$

from which results

(187) $$\left[\begin{array}{cc} {\bar{\varepsilon }}_{22} & {\bar{\varepsilon }}_{33}\\ {\bar{\varepsilon }}_{33} & {\bar{\varepsilon }}_{22} \end{array}\right]\left\{\begin{array}{c} C_{22}\\ C_{23} \end{array}\right\}=\left\{\begin{array}{c} {\bar{\sigma }}_{22}\\ {\bar{\sigma }}_{33} \end{array}\right\}$$

and

(188) $$\left\{\begin{array}{c} C_{22}\\ C_{23} \end{array}\right\}=\frac{1}{{\bar{\varepsilon }}_{22}^{2}-{\bar{\varepsilon }}_{33}^{2}}\left[\begin{array}{cc} {\bar{\varepsilon }}_{22} & -{\bar{\varepsilon }}_{33}\\ -{\bar{\varepsilon }}_{33} & {\bar{\varepsilon }}_{22} \end{array}\right]\left\{\begin{array}{c} {\bar{\sigma }}_{22}\\ {\bar{\sigma }}_{33} \end{array}\right\}$$

This results in the following:

(189) $$C_{22}=\frac{{\bar{\sigma }}_{22}{\bar{\varepsilon }}_{22}-{\bar{\sigma }}_{33}{\bar{\varepsilon }}_{33}}{{\bar{\varepsilon }}_{22}^{2}-{\bar{\varepsilon }}_{33}^{2}}\;;\;C_{23}=\frac{{\bar{\sigma }}_{33}{\bar{\varepsilon }}_{22}-{\bar{\sigma }}_{22}{\bar{\varepsilon }}_{33}}{{\bar{\varepsilon }}_{22}^{2}-{\bar{\varepsilon }}_{33}^{2}}\;$$

For $C_{12}$ and $C_{66}$,

(190) $$C_{12}=\frac{{\bar{\sigma }}_{11}}{{\bar{\varepsilon }}_{22}+{\bar{\varepsilon }}_{33}}\;;\;C_{66}=\frac{{\bar{\tau }}_{23}}{{\bar{\gamma }}_{23}}\;$$

To determine the bulk modulus $K_{23}$ is used in the relation:

(191) $$K_{23}=\frac{C_{22}+C_{33}}{2}=\frac{{\bar{\sigma }}_{22}+{\bar{\sigma }}_{33}}{2\left({\bar{\varepsilon }}_{22}+{\bar{\varepsilon }}_{33}\right)}\;.$$

The longitudinal Poisson’s ratio is calculated via the following relation:

(192) $$\nu_{1}=\nu_{21}=\nu_{31}=\frac{1}{2}{\left(\frac{C_{11}-E_{11}}{K_{23}}\right)}^{1/2}=\frac{C_{12}}{C_{22}+C_{33}}=\frac{{\bar{\sigma }}_{11}}{\left({\bar{\sigma }}_{22}+{\bar{\sigma }}_{33}\right)}\;.$$

and the shear modulus

(193) $$G_{23}=\frac{C_{22}-C_{33}}{2}=\frac{{\bar{\sigma }}_{22}-{\bar{\sigma }}_{33}}{2\left({\bar{\varepsilon }}_{22}-{\bar{\varepsilon }}_{33}\right)}\;.$$

or from

(194) $$G_{23}=C_{66}=\frac{{\bar{\sigma }}_{23}}{2{\bar{\varepsilon }}_{23}}\;.$$

By introducing the following parameter,

(195) $$\psi =1+\frac{4\nu_{1}^{2}K_{23}}{E_{11}}\;,$$

the transverse moduli and Poisson’s ratio are obtained using the following relations:

(196) $$E_{22}=E_{33}=\frac{4G_{23}K_{23}}{K_{23}+\psi G_{23}}\;$$

and

(197) $$\nu_{23}=\frac{K_{23}-\psi G_{23}}{K_{23}+\psi G_{23}}\;$$

As such, the expressions for $E_{11},\;E_{22}=E_{33},\;\nu_{12}=\nu_{13},\;\nu_{23},\;G_{23},K_{23}$ were determined. From

(198) $$C_{22}+C_{33}=2K_{23}\;;\;C_{22}-C_{33}=2G_{23}\;$$

one may obtain

(199) $$C_{22}=K_{23}+G_{23}\;;\;C_{23}=K_{23}-G_{23}\;.$$

Recall that

(200) $$C_{44}=G_{1}=G_{12}=G_{13};\;C_{12}=\nu_{1}\left(C_{22}+C_{23}\right)=2\nu_{1}K_{23}\;$$

and

(201) $$C_{11}=E_{11}+\frac{2C_{12}^{2}}{C_{22}+C_{23}}=E_{11}+4\nu_{1}^{2}K_{23}=\psi E_{11}\;.$$

In a similar way, FEM was used to determine the average stresses and strains in a 3D elastic solid. These are:${\bar{\sigma }}_{11},{\bar{\sigma }}_{22},{\bar{\sigma }}_{33},{\bar{\sigma }}_{12}={\bar{\tau }}_{12},{\bar{\sigma }}_{23}={\bar{\tau }}_{23},{\bar{\sigma }}_{31}={\bar{\tau }}_{31},$ ${\bar{\varepsilon }}_{11},{\bar{\varepsilon }}_{22},{\bar{\varepsilon }}_{33},{\bar{\varepsilon }}_{12}=1/2\;\gamma_{12},$ ${\bar{\varepsilon }}_{23}=1/2\;\gamma_{23},{\bar{\varepsilon }}_{31}=1/2\;\gamma_{31}$. The general Hooke’s Law can be written as follows:

(202) $${\bar{\sigma }}_{11}=C_{11}{\bar{\varepsilon }}_{11}+C_{12}{\bar{\varepsilon }}_{22}+C_{12}{\bar{\varepsilon }}_{33}\;{\bar{\sigma }}_{22}=C_{12}{\bar{\varepsilon }}_{11}+C_{22}{\bar{\varepsilon }}_{22}+C_{23}{\bar{\varepsilon }}_{33}\;{\bar{\sigma }}_{33}=C_{12}{\bar{\varepsilon }}_{11}+C_{23}{\bar{\varepsilon }}_{22}+C_{22}{\bar{\varepsilon }}_{33}\;{\bar{\sigma }}_{23}={\bar{\tau }}_{23}=\left(C_{11}-C_{23}\right){\bar{\varepsilon }}_{23}=\frac{1}{2}\left(C_{11}-C_{23}\right){\bar{\gamma }}_{23}\;{\bar{\sigma }}_{31}={\bar{\tau }}_{31}=2C_{44}{\bar{\varepsilon }}_{31}=C_{66}{\bar{\gamma }}_{31}\;{\bar{\sigma }}_{12}={\bar{\tau }}_{12}=2C_{44}{\bar{\varepsilon }}_{12}=C_{66}{\bar{\gamma }}_{12}$$

From the last part of Equation (202), we can obtain

(203) $$C_{44}=\frac{{\bar{\sigma }}_{12}}{2\;{\bar{\varepsilon }}_{12}}=\frac{{\bar{\tau }}_{12}}{{\bar{\gamma }}_{12}}=G_{12}=G_{13}=G_{1}$$

Equation (202) yields

(204) $${\bar{\sigma }}_{22}-{\bar{\sigma }}_{33}=\left(C_{22}-C_{33}\right)\left({\bar{\varepsilon }}_{22}-{\bar{\varepsilon }}_{33}\right)$$

The law of mixture offers us

(205) $$E_{11}=E_{f}v_{f}+E_{m}v_{m}$$

Using Equation (205), one can replace the redundant fourth relation from Equation (201) with

(206) $$E_{11}=C_{11}-\frac{2C_{12}^{2}}{C_{22}+C_{23}}$$

The addition of the second and third equations in Equation (202) yields

(207) $${\bar{\sigma }}_{22}+{\bar{\sigma }}_{33}-\frac{2C_{12}{\bar{\varepsilon }}_{11}}{{\bar{\varepsilon }}_{22}+{\bar{\varepsilon }}_{33}}=C_{22}+C_{23}$$

From the first part of Equation (202), one can show that

(208) $$C_{11}=\frac{{\bar{\sigma }}_{11}-C_{12}\left({\bar{\varepsilon }}_{22}+{\bar{\varepsilon }}_{33}\right)}{{\bar{\varepsilon }}_{11}}$$

The substitution of Equation (208) into $E_{11}$ yields

(209) $$E_{11}=\frac{{\bar{\sigma }}_{11}}{{\bar{\varepsilon }}_{11}}+C_{12}\frac{{\bar{\varepsilon }}_{22}+{\bar{\varepsilon }}_{33}}{{\bar{\varepsilon }}_{11}}-\frac{2C_{12}^{2}\left({\bar{\varepsilon }}_{22}+{\bar{\varepsilon }}_{33}\right)}{\left({\bar{\sigma }}_{22}+{\bar{\sigma }}_{33}-2C_{12}{\bar{\varepsilon }}_{11}\right)}$$

from which it is possible to compute $C_{12}$.

**Figure 8:**
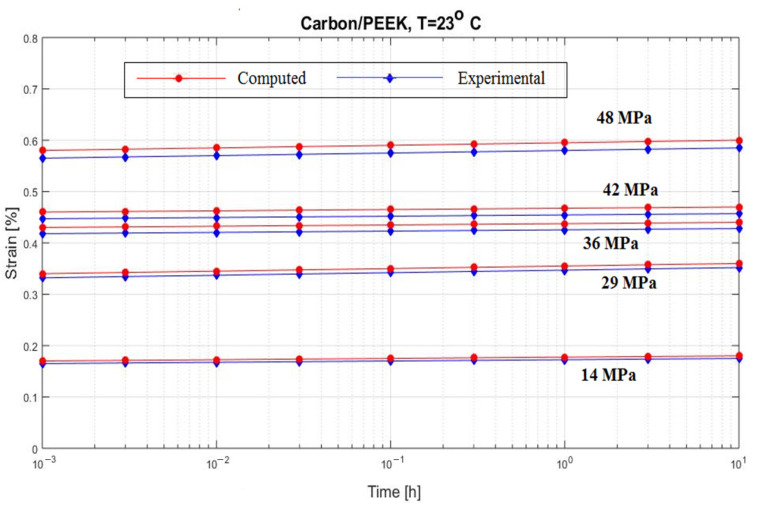
Creep response of a carbon/epoxy {90}_4s_ at T = 23 °C.

**Figure 9:**
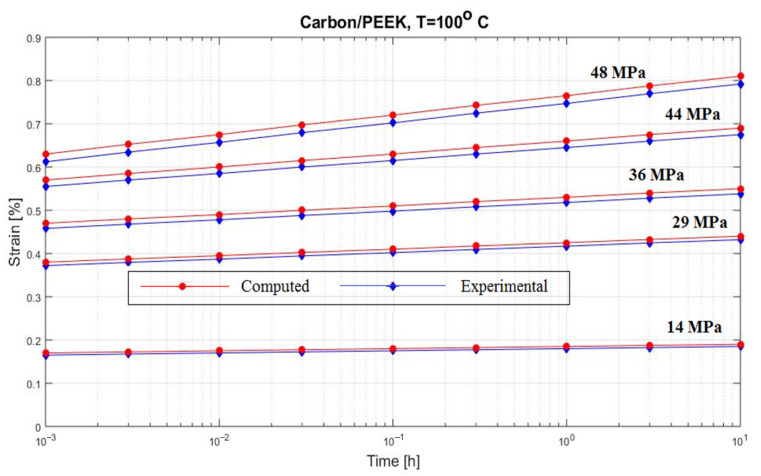
Creep response of a carbon/epoxy {90}_4s_ at T = 100 °C.

Figure 8 and Figure 9 present two creep curves for a composite carbon/epoxy at two different temperatures [56,57].

## 6. Conclusions

The method presented in this paper proves to be a calculus method suitable for obtaining the general mechanical constants of a multiphase composite material. The material constants required by designers are obtained using the average of the values obtained by applying FEM. The results obtained experimentally verified the models proposed by different researchers. The tests and measurements conducted here show a good concordance between the results obtained using the proposed models and the experimental verifications. Thus, FEM proves to be a powerful tool for determining the engineering constants of composite materials. Compared to the methods described in the other sections, this method proves to be a useful and relatively simple means of identifying the constitutive laws. The results were also applied to a study of the creep behavior of a composite material. This case is more complicated because, in the case of creep phenomena, the influences of temperature prove to be nonlinear. All the presented models can replace expensive methods of determining the engineering constants of a viscoelastic material by experimental measurements with calculation-based methods.

This review focuses on the behavior of unidirectional fiber composites.

## Figures and Tables

**Table 1:** Average values of stress and strain (Case 1).

$\bar{\sigma }$	Fiber	Matrix	RUC	$\bar{\varepsilon }$	Fiber	Matrix	RUC
${\bar{\sigma }}_{22}$	0.146 × 10^3^	0.122 × 10^3^	0.138 × 10^3^	${\bar{\varepsilon }}_{22}$	0.16 × 10^0^	0.263 × 10^1^	0.106 × 10^1^
${\bar{\sigma }}_{33}$	0.71 × 10^0^	−0.906 × 10^0^	0.118 × 10^0^	${\bar{\varepsilon }}_{33}$	−0.445 × 10^−1^	−0.137 × 10^1^	−0.529 × 10^0^
${\bar{\sigma }}_{11}$	0.324 × 10^2^	0.413 × 10^3^	0.357 × 10^2^	${\bar{\varepsilon }}_{11}$	0.0 × 10^0^	0.0 × 10^0^	0.0 × 10^0^
${\bar{\sigma }}_{23}$	0.194 × 10^−4^	0.412 × 10^2^	0.359 × 10^−4^	${\bar{\varepsilon }}_{23}$	0.539 × 10^−7^	0.447 × 10^−5^	0.167 × 10^−5^

**Table 2:** Computed values of elastic moduli (Case 1).

Modulus [MPa]	Matrix	Fiber	Average
$E_{11}$	4140.0	86,900.0	56,278.0
$E_{23}=E_{13}$	4140.0	86,899.0	12,741.0
$\nu_{1}$	0.34	0.22	0.259
$\nu_{23}$	0.34	0.22	0.475
$G_{23}$	1544.0	35,614.7	4318.2
$K_{23}$	4827.4	63,597.7	12,886.2

**Table 3:** Average values of stress and strain (Case 2).

$\bar{\sigma }$	Fiber	Matrix	RUC	$\bar{\varepsilon }$	Fiber	Matrix	RUC
${\bar{\sigma }}_{22}$	0.147 × 10^3^	0.122 × 10^3^	0.138 × 10^3^	${\bar{\varepsilon }}_{22}$	0.134 × 10^0^	0.183 × 10^1^	0.757 × 10^0^
${\bar{\sigma }}_{33}$	0.857 × 10^2^	0.702 × 10^2^	0.801 × 10^2^	${\bar{\varepsilon }}_{33}$	0.485 × 10^−1^	0.157 × 10^0^	0.881 × 10^−1^
${\bar{\sigma }}_{11}$	0.512 × 10^2^	0.653 × 10^2^	0.564 × 10^2^	${\bar{\varepsilon }}_{11}$	0.0 × 10^0^	0.0 × 10^0^	0.0 × 10^0^
${\bar{\sigma }}_{23}$	0.992 × 10^−5^	0.322 × 10^−4^	0.181 × 10^−4^	${\bar{\varepsilon }}_{23}$	0.306 × 10^−7^	0.190 × 10^−5^	0.717 × 10^−6^

**Table 4:** Computed values of the elastic moduli (Case 2).

Modulus	Matrix	Fiber	Average
$E_{11}$	4140.0	86,900.0	56,278.0
$E_{23}=E_{13}$	4140.0	86,900.0	12,741.0
$\nu_{1}$	0.34	0.22	0.259
$\nu_{23}$	0.34	0.22	0.475
$G_{23}$	1544.0	35,614.8	4318.2
$K_{23}$	4827.4	63,597.8	12,886.2

**Table 5:** Average values of stress and strain (Case 3).

$\bar{\sigma }$	Fiber	Matrix	RUC	$\bar{\varepsilon }$	Fiber	Matrix	RUC
${\bar{\sigma }}_{22}$	0.147 × 10^3^	0.123 × 10^3^	0.138 × 10^3^	${\bar{\varepsilon }}_{22}$	0.160 × 10^0^	0.263 × 10^1^	0.106 × 10^−1^
${\bar{\sigma }}_{33}$	0.644 × 10^0^	−0.983 × 10^0^	0.049 × 10^−1^	${\bar{\varepsilon }}_{33}$	−0.446 × 10^−1^	−0.137 × 10^1^	0.530 × 10^0^
${\bar{\sigma }}_{11}$	0.324 × 10^2^	0.414 × 10^2^	0.357 × 10^2^	${\bar{\varepsilon }}_{11}$	0.0 × 10^0^	0.0 × 10^0^	0.0 × 10^0^
${\bar{\sigma }}_{23}$	0.899 × 10^−5^	−0.979 × 10^−4^	−0.301 × 10^−4^	${\bar{\varepsilon }}_{23}$	0.258 × 10^−7^	−0.638 × 10^−5^	0.232 × 10^−5^

**Table 6:** Computed values of the elastic moduli (Case 3).

Modulus	Matrix	Fiber	Average
$E_{11}$	4140.0	86,900.0	56,279.0
$E_{23}=E_{13}$	4140.0	86,899.0	12,754.0
$\nu_{1}$	0.34	0.22	0.259
$\nu_{23}$	0.34	0.22	0.475
$G_{23}$	1544.0	35,614.7	4322.2
$K_{23}$	4827.4	63,597.7	12,900.8

**Table 7:** Finite element models and associated boundary conditions (BCs).

Case	Model	B.C. (x_2_ Direction)	B.C. (x_3_ Direction)
1	Model 1-a	*p_x_* = 137.90 (MPa)	*p_y_* = 0.00 (MPa)
2	Model 1-b	*p_x_* = 137.90 (MPa)	*p_y_* = 80.0 (MPa)
3	Model 2-a	*p_x_* = 137.90 (MPa)	*p_y_* = 0.00 (MPa)
4	Model 2-b	*u_x_* = 0.01 (mm)	*u_y_* = 0.01 (mm)
5	Model 2-c	*u_x_* = 0.01 (mm)	*u_y_* = 0.01 (mm)

## Data Availability

Not applicable.

## Appendix A

Eshelby’s tensor for an elliptic cylinder:

(A1) $$P_{2222}=\frac{1}{2\left(1-v_{m}\right)}\left[\frac{1+2\alpha }{{\left(1+\alpha \right)}^{2}}+\frac{1-2v_{m}}{1+\alpha }\right]\;P_{3333}=\frac{\alpha }{2\left(1-v_{m}\right)}\left[\frac{\alpha +2}{{\left(1+\alpha \right)}^{2}}+\frac{1-2v_{m}}{1+\alpha }\right]\;P_{2211}=\frac{v_{m}}{1-v_{m}}\frac{1}{1+\alpha }\;P_{2233}=\frac{1}{2\left(1-v_{m}\right)}\left[\frac{1}{{\left(1+\alpha \right)}^{2}}+\frac{1-2v_{m}}{1+\alpha }\right]\;P_{3311}=\frac{v_{m}}{1-v_{m}}\frac{\alpha }{1+\alpha }\;P_{3322}=\frac{\alpha }{2\left(1-v_{m}\right)}\left[\frac{\alpha }{{\left(1+\alpha \right)}^{2}}+\frac{1-2v_{m}}{1+\alpha }\right]\;P_{1212}=\frac{1}{2\left(1+\alpha \right)}\;P_{1313}=\frac{\alpha }{2\left(1+\alpha \right)}\;P_{2323}=\frac{1}{4\left(1-v_{m}\right)}\left[\frac{1+\alpha^{2}}{{\left(1+\alpha \right)}^{2}}+\left(1-2v_{m}\right)\right]$$

and

(A2) $$P_{1111}=0\;;\;P_{1122}=0\;;\;P_{1133}=0$$

where all other $P_{ijkl}=0$. Relation (9) presents the link between $\varepsilon_{ij}^{0}$ and $\varepsilon_{ij}^{*}$. It must use the following relations (see Reference [25]):

(A3) $$\left[\begin{array}{ccc} M_{1} & M_{2} & M_{3}\\ M_{4} & M_{5} & M_{6}\\ M_{7} & M_{8} & M_{9} \end{array}\right]\left\{\begin{array}{c} \varepsilon_{11}^{*}\\ \varepsilon_{22}^{*}\\ \varepsilon_{33}^{*} \end{array}\right\}+\left[\begin{array}{ccc} N_{1} & 1 & 1\\ 1 & N_{2} & 1\\ 1 & 1 & N_{3} \end{array}\right]\left\{\begin{array}{c} \varepsilon_{11}^{0}\\ \varepsilon_{22}^{0}\\ \varepsilon_{33}^{0} \end{array}\right\}=0$$

where

(A4) $$M_{1}=v_{f}N_{1}+N_{2}+v_{m}\left(P_{2211}+P_{3311}\right);\;M_{2}=v_{f}+N_{3}+v_{m}\left(P_{2222}+P_{3322}\right);\;M_{3}=v_{f}+N_{3}+v_{m}\left(P_{2233}+P_{3333}\right);\;M_{4}=v_{f}+N_{3}+v_{m}\left(P_{2211}+P_{3311}\right);\;M_{5}=v_{f}N_{1}+N_{2}+v_{m}\left(N_{1}P_{2222}+P_{3322}\right);\;M_{6}=v_{f}+N_{3}+v_{m}\left(P_{2233}+P_{3333}\right);\;M_{7}=v_{f}+N_{3}+v_{m}\left(N_{1}P_{3311}+P_{2211}\right);\;M_{8}=v_{f}+N_{3}+v_{m}\left(N_{1}P_{3322}+P_{2222}\right);\;M_{9}=v_{f}N_{1}+N_{2}+v_{m}\left(P_{3333}+P_{2233}\right)$$

and

(A5) $$N_{1}=1+2\left(G_{f}-G_{m}\right)/\left(\lambda_{f}-\lambda_{m}\right);\;N_{2}=\left(\lambda_{m}+2G_{m}\right)/\left(\lambda_{f}-\lambda_{m}\right);\;N_{3}=\lambda_{m}/\left(\lambda_{f}-\lambda_{m}\right)$$

where $\lambda_{f}$ and $\lambda_{m}$ are the Lamé constants (for the fiber and the matrix).

From (A3), we obtain the following:

(A6) $$\left\{\begin{array}{c} \varepsilon_{11}^{*}\\ \varepsilon_{22}^{*}\\ \varepsilon_{33}^{*} \end{array}\right\}=\frac{1}{A}\left[\begin{array}{ccc} A_{11} & A_{12} & A_{13}\\ A_{21} & A_{22} & A_{23}\\ A_{31} & A_{32} & A_{33} \end{array}\right]\left\{\begin{array}{c} \varepsilon_{11}^{0}\\ \varepsilon_{22}^{0}\\ \varepsilon_{33}^{0} \end{array}\right\}$$

(A7) $$A_{11}=A\cdot a_{11}=N_{1}\left(M_{6}M_{8}-M_{5}M_{9}\right)+M_{3}\left(M_{5}-M_{8}\right)+M_{2}\left(M_{9}-M_{6}\right);\;A_{12}=A\cdot a_{12}=N_{1}\left(M_{2}M_{9}-M_{3}M_{8}\right)+M_{6}\left(M_{8}-M_{2}\right)+M_{5}\left(M_{3}-M_{9}\right);\;A_{13}=A\cdot a_{13}=N_{1}\left(M_{3}M_{5}-M_{2}M_{6}\right)+M_{8}\left(M_{6}-M_{3}\right)+M_{9}\left(M_{2}-M_{5}\right);\;A_{21}=A\cdot a_{21}=N_{1}\left(M_{4}M_{9}-M_{6}M_{7}\right)+M_{1}\left(M_{6}-M_{9}\right)+M_{3}\left(M_{7}-M_{4}\right);\;A_{22}=A\cdot a_{22}=N_{1}\left(M_{3}M_{7}-M_{1}M_{9}\right)+M_{4}\left(M_{9}-M_{3}\right)+M_{6}\left(M_{1}-M_{7}\right);\;A_{23}=A\cdot a_{23}=N_{1}\left(M_{1}M_{6}-M_{3}M_{4}\right)+M_{9}\left(M_{4}-M_{1}\right)+M_{7}\left(M_{3}-M_{6}\right);\;A_{31}=A\cdot a_{31}=N_{1}\left(M_{5}M_{7}-M_{4}M_{8}\right)+M_{2}\left(M_{4}-M_{7}\right)+M_{1}\left(M_{8}-M_{5}\right);\;A_{32}=A\cdot a_{32}=N_{1}\left(M_{1}M_{8}-M_{2}M_{7}\right)+M_{5}\left(M_{7}-M_{1}\right)+M_{4}\left(M_{2}-M_{8}\right);\;A_{33}=A\cdot a_{33}=N_{1}\left(M_{2}M_{4}-M_{1}M_{5}\right)+M_{7}\left(M_{5}-M_{2}\right)+M_{8}\left(M_{1}-M_{4}\right)$$
